# Grouping of nanomaterials to read-across hazard endpoints: from data collection to assessment of the grouping hypothesis by application of chemoinformatic techniques

**DOI:** 10.1186/s12989-018-0273-1

**Published:** 2018-09-24

**Authors:** L. Lamon, D. Asturiol, A. Richarz, E. Joossens, R. Graepel, K. Aschberger, A. Worth

**Affiliations:** European Commission, Joint Research Centre, Ispra, Varese Italy

**Keywords:** Grouping, Nanomaterials, Read-across, Nano-TiO_2_, Comet assay, RAAF, REACH, Hazard, Chemoinformatics

## Abstract

**Background:**

An increasing number of manufactured nanomaterials (NMs) are being used in industrial products and need to be registered under the REACH legislation. The hazard characterisation of all these forms is not only technically challenging but resource and time demanding. The use of non-testing strategies like read-across is deemed essential to assure the assessment of all NMs in due time and at lower cost. The fact that read-across is based on the structural similarity of substances represents an additional difficulty for NMs as in general their structure is not unequivocally defined. In such a scenario, the identification of physicochemical properties affecting the hazard potential of NMs is crucial to define a grouping hypothesis and predict the toxicological hazards of similar NMs. In order to promote the read-across of NMs, ECHA has recently published “Recommendations for nanomaterials applicable to the guidance on QSARs and Grouping”, but no practical examples were provided in the document. Due to the lack of publicly available data and the inherent difficulties of reading-across NMs, only a few examples of read-across of NMs can be found in the literature. This manuscript presents the first case study of the practical process of grouping and read-across of NMs following the workflow proposed by ECHA.

**Methods:**

The workflow proposed by ECHA was used and slightly modified to present the read-across case study. The Read-Across Assessment Framework (RAAF) was used to evaluate the uncertainties of a read-across within NMs. Chemoinformatic techniques were used to support the grouping hypothesis and identify key physicochemical properties.

**Results:**

A dataset of 6 nanoforms of TiO_2_ with more than 100 physicochemical properties each was collected. In vitro comet assay result was selected as the endpoint to read-across due to data availability. A correlation between the presence of coating or large amounts of impurities and negative comet assay results was observed.

**Conclusion:**

The workflow proposed by ECHA to read-across NMs was applied successfully. Chemoinformatic techniques were shown to provide key evidence for the assessment of the grouping hypothesis and the definition of similar NMs. The RAAF was found to be applicable to NMs.

**Electronic supplementary material:**

The online version of this article (10.1186/s12989-018-0273-1) contains supplementary material, which is available to authorized users.

## Background

Chemicals safety assessment is addressed in Europe by the Regulation (EC) No 1907/2006 concerning the Registration, Evaluation, Authorisation and Restriction of Chemicals (REACH) [1] which requires companies to assess the risks posed by marketed chemicals. This implies the generation of toxicological data as it is required in risk assessment to address any identified hazard. It is stated in the REACH legislation that all available in vitro, in vivo and historical human data, data from valid (Q)SARs and data from structurally related similar substances (read-across approach), must be assessed before carrying out any test.

The use of non-testing strategies like read-across is key for nanomaterials (NMs) as estimations suggest that between 500 and 2000 NMs with < 10 nanoforms^1^ per NM type are/will be manufactured or imported in Europe in quantities greater than 1 t/annum [2, 3].

Read-across is regarded as a technique for predicting endpoint information for one or more substances (target substance(s)) by using data from the same endpoint from (an)other substance(s) provided that these substances are similar, i.e. have similar physicochemical, toxicological and ecotoxicological properties, or follow a regular pattern as a result of structural similarity that allows them to be considered a group (REACH Annex XI). The identification of structurally similar substances is more challenging for NMs than regular chemicals because NMs do not have a uniquely defined structure. The European Chemicals Agency (ECHA) has recently released guidance on how to justify grouping for read-across between nanoforms of the same substance [4]. This guidance proposes a revised version of a strategy presented earlier [5] and considers properties beyond chemical composition (e.g. aspect ratio, particle size, shape, or solubility), and reaffirms the similarity rules from REACH Annex XI for NMs.

In spite of the efforts put to favour the use of read-across between nanoforms [5–10] only a few examples of read-across for NMs are found in the literature. One of this examples corresponds to the cytotoxicity of metal oxides for E. coli and HaCaT cell line (human keratinocytes), which uses physicochemical properties like the enthalpy of formation of the metal oxide nanocluster and Mulliken’s electronegativity to determine similarity [11]. Another study proposes a NM ranking based on solubility and band gap [12]. These case studies are illustrative for the fact that available studies mostly use physicochemical properties that are not specific to NMs to support grouping based on similarity. Other examples available in the literature are exemplified by the application of the DF4nanoGrouping framework to 24 NM of different types (carbon based, metal and metal oxide, silica, organic) [13], which groups NM into 4 subgroups (soluble, biopersistent, passive, and active) for further read-across. This framework takes into consideration NM-specific physicochemical properties like particle morphology and composition, dissolution rate, surface reactivity, dispersibility.

In this manuscript, we present a case study of grouping and read-across of TiO_2_ nanoforms where we apply a simplified version of the grouping framework proposed by ECHA to predict the in vitro comet assay results of the target substances. One key step in read-across is the determination of the physicochemical properties that define the groups and similarities between analogues of the same category, which was achieved with the help of chemoinformatic techniques such as hierarchical clustering (HC), principal component analysis (PCA), and random forest variable selection. Evaluation of uncertainties in the similarity and read-across justifications is an important part of a read-across exercise. ECHA developed the Read-Across Assessment framework (RAAF) as guidance for systematic analysis of uncertainties in read-across justifications submitted for REACH. In this case study, the confidence in the read-across argumentation was evaluated following the RAAF also in view of assessing whether the RAAF is, with the given scenarios, applicable to NMs. Considering that the RAAF is based on chemical structural similarity and consistent with the REACH definition of similarity for read-across, it is expected that the main difficulties for its application to NMs will be related to their characterisation and to the properties associated to the toxicological effect.

## Methods

### Workflow for grouping and read-across

**Fig. 1:**
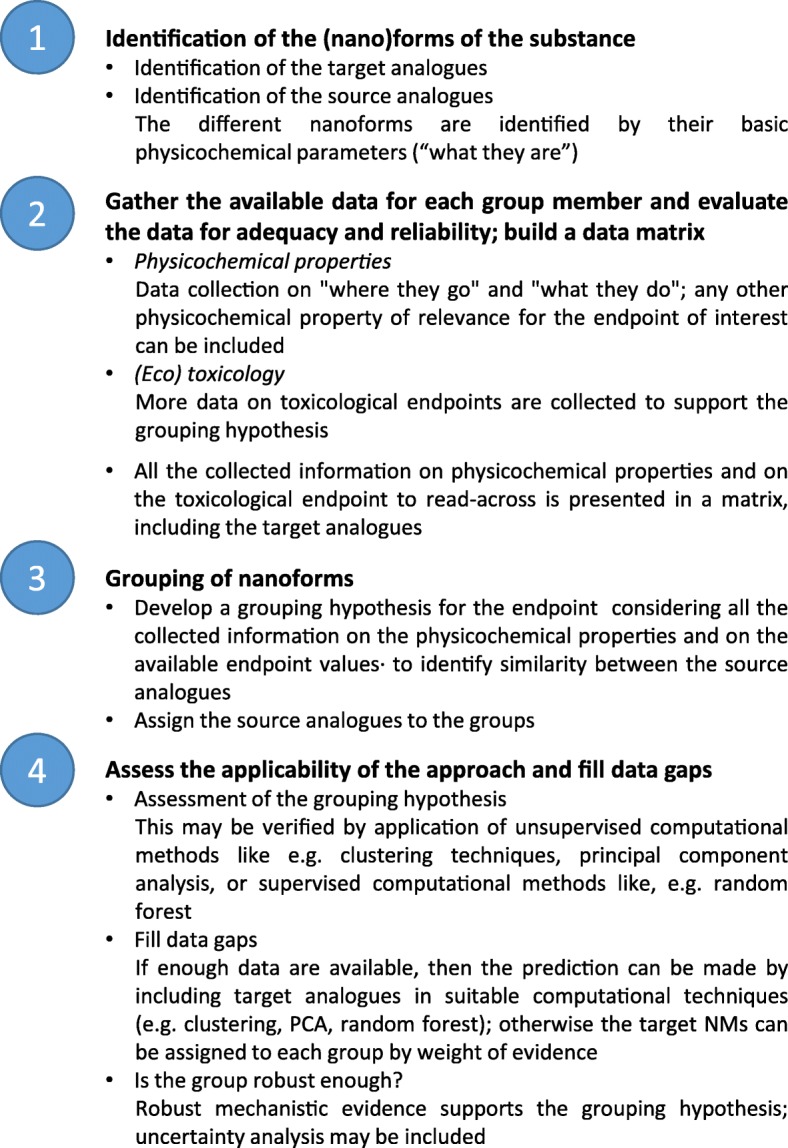
Framework for grouping and read-across for reporting the nano-TiO_2_ case study. A simplified version from the framework proposed by ECHA [4]

The present case study follows a simplified version of the workflow proposed by ECHA [4], as illustrated in Fig. 1.

Step 1 of the framework corresponds to the *identification of the nanoforms of the substance* including source (analogues) and target substances, i.e. “what they are” [14], where NMs are identified through properties like composition, impurities, surface chemistry, size, shape. Step 2 on *gathering of data for each group member and evaluate the data for adequacy and reliability* consists in collecting data for each analogue on “where they go”, including properties like solubility, hydrophobicity, zeta potential, size distribution, dispersibility, dustiness, and “what they do”, including properties related to redox activity. A matrix reports the collected information for analysis. Step 3 *grouping of nanoforms* consists in the analysis to identify similarities between analogues and to build the grouping hypothesis. Step 4 *assess the applicability of the approach, and fill data gaps* consists in the justification of the grouping hypothesis by means of chemoinformatic techniques and the read-across prediction; this step involves also an assessment of the robustness of the grouping hypothesis by supporting it with mechanistic evidence and uncertainty analysis.

In the case of NMs, the definition of analogues is not as straightforward as for e.g. organic chemicals, because the influence that the different properties (e.g. size, coating, composition, or solubility) can have on their behaviour (activity) is not yet well understood. If enough data is available, chemoinformatics may also be used to identify the relevant properties for a specific endpoint.

### Computational methods

A set of statistical methods often used in chemoinformatics were applied using R 3.2.5 [15] to identify the most relevant (physicochemical) properties to determine similarity between analogues and support the grouping hypothesis. These techniques were:

1. Hierarchical clustering (HC) [16]: was applied to identify possible clusters or groups of analogues in the dataset, i.e. similar NMs
2. Principal component analysis (PCA) [17]: was applied to determine the physicochemical properties that differentiate the NMs and to observe possible clusters of NM and properties
3. Random forest variable selection [18]: was applied to determine the most relevant properties in predicting in vitro comet assay results. Unlike hierarchical clustering and PCA this is a supervised technique and, therefore, makes use of physicochemical properties to *predict* a given outcome, which in this case was genotoxicity as determined by the comet assay.

### Data treatment

Our initial dataset on toxicological endpoints was collected from the OECD dossier on TiO_2_ [19] that, although not aimed specifically at hazard assessment, is considered an updated NMs data repository. This toxicological dataset was expanded for the selected endpoint to be read-across by searching available studies in the literature. The final dataset consisted mainly of tests carried out within the Nanogenotox Joint Action [20]. A reliability assessment of the collected studies was performed according to the criteria defined by the French agency for food, environmental and occupational health and safety (ANSES) [21], which states that reliable studies must contain:

1. NMs characterisation (at least size, crystallinity and coating) and a description of the dispersed materials (particle size distribution, zeta potential, polydispersity index)
2. Observed NM uptake and/or non-cytotoxicity
3. Positive and negative controls as well as replicates

Due to the lack of standard operating procedures (SOPs) for NMs, the collected data for nano-TiO_2_ was found to contain the same measures with different techniques (e.g. Dynamic Light Scattering for particle size distribution and Transmission Electron microscope for particle size), data measured in different solvents (e.g. MilliQ water, Fetal Bovine Serum, Phosphate-Buffered Saline), or with different pre-treatments (e.g. not sonicated, 1 min sonication with tip sonication, 20 min bath sonication). In such a situation, two options can be considered: a) each technique, instrument, media, and pre-treatment is considered as a different property or b) data from different origins is merged into a common value. Both options present advantages and disadvantages. Keeping each value as a different measure leads to a dataset with a number of data gaps, which is unusable for modelling or read-across as the properties are not considered comparable. Therefore, it becomes almost impossible to compare two substances. In order to avoid this scenario, the data obtained from different sources was merged. A detailed explanation of how the data was merged for each property can be found in Section 1.2 of the Additional file 1.

### Read-across assessment framework

The ECHA RAAF [22] was used as guidance for a structured evaluation of uncertainties in the read-across argumentation. It distinguishes six scenarios defined by the read-across approach taken (analogue or category approach), whether the effect is caused by identical or different compounds for the source(s) and target(s) – which can be either the parent or metabolites formed by biotransformation, respectively – and whether the predicted property is following a trend in the category or not changing across source structures. For each scenario a set of Assessment Elements (AEs), comprising multiple considerations and questions, has to be addressed. They evaluate amongst others the similarity hypothesis, availability and quality of data, and the postulated mechanism of toxicity. The outcome of the analysis and conclusions on the scientific robustness and validity of the read-across justification are scored with Assessment Options, i.e. scores from one to five indicating whether the information provided is not acceptable at all (1), or in its current form (2), or acceptable with just sufficient (3), medium (4) or high (5) confidence.

From the six RAAF scenarios, scenario 6 was chosen as best describing the present case of nano-TiO_2_ read-across. It corresponds to a category approach, with different compounds (i.e. nanoforms) considered to have the same type of effect, and no variations in the effect, i.e. the comet assay result is either positive or negative, but has no varying potency following a trend. The read-across hypothesis is judged via assessment elements C.1-C.6, common to all RAAF scenarios, and 6.1–6.5 as specific AEs for scenario 6.

## Results

This section is structured following the workflow of Fig. 1.

### Step 1: Identification of the (nano)forms of the substance

According to ECHA's guidance [4], and following the workflow presented in Fig. 1, analogues were identified through the following physicochemical parameters (“what they are”): chemical composition, crystalline structure, impurities, surface chemistry, particle size, shape, surface area, and porosity.

#### Identification of the target analogues

According to the physicochemical properties [23] (see Table 1) the target materials consist of TiO_2_ nanopowders of rutile (TiO_2_ R) and anatase (TiO_2_ A), respectively. TiO_2_ A has a specific surface area of 149 m^2^/g, is uncoated, and 99.5% w/w pure; while TiO_2_ R has a specific surface area of 177 m^2^/g, is coated, and 87% w/w pure. According to the producer, TiO_2_ R nano may contain up to 5% w/w of SiO_2_ as surface coating (see Sigma-Aldrich ref. 637,262).

**Table 1:** Physicochemical properties (“what they are”) of the source and target analogues [19, 23]

Property	NM-100	NM-101	NM-102	NM-103	NM-104	NM-105	TiO_2_ R nano	TiO_2_ A nano
Crystal type	Anatase	Anatase	Anatase	Rutile	Rutile	83% anatase 17% rutile	Rutile	Anatase
Total non-TiO_2_ content including coating and impurities (% w/w)	1.5	9	5	11	11	0.11	13	0.50
Surface chemistry (as declared by manufacturer)^f^	uncoated	uncoated	uncoated	Al_2_O_3_, (C_2_H_6_OSi)_n_and C_6_H_16_O_2_Si	Al_2_O_3_, (C_2_H_6_OSi)_n_and C_3_H_8_O_3_	uncoated	SiO_2_ (< 5%) Na_2_SO_4_ ${\mathrm{SO}}_4^{-2}$	uncoated
Surface coating (% w/w)	0	0	0	8	8	0	11	0
Primary particle diameter (TEM) (nm)	93 ± 23	5 ± 1	22 ± 10	24 ± 2	24 ± 2	20 ± 3	10 nm diameter 62 nm length	14
Crystallite size (XRD) (nm)^a^	117 ± 40	7 ± 2	24 ± 5	24 ± 4	25 ± 4	22 ± 5	–	–
Particle Size Distribution (nm)	210 ± 10^b^	278^b^	440 ± 37^b^	135 ± 25^b^	145 ± 35^b^	177 ± 39^b^	125^c^	145^c^
Shape	Spheroidal	Spheroidal	Spheroidal	Spheroidal	Spheroidal	Spheroidal	Rod	Sphere
Aspect ratio	1.53	1.53	1.53	1.7	1.53	1.36	–	–
Specific surface area (m^2^/g)	9^d^	242 ± 73^d^	77 ± 10^d^	54 ± 4^d^	54 ± 2^d^	47 ± 0.5^d^	177^e^	149^e^
Total pore volume (ml/g)	0.0324	0.319	0.2996	0.2616	0.1935	0.1937	–	–

^a^values averaged from different instruments and principles (Peak fit, TOPAS, Fullprof, Scherrer eq., TOPAS, IB, TOPAS FWHM)

^b^value from DLS

^c^values averaged from ICP-MS and DLS experiments

^d^values averaged from SAXS/USAXS and BET

^e^value from BET

^f^(C_2_H_6_OSi)_n_ indicates presence of dimethicone, C_6_H_16_O_2_Si of dimethoxydimethylsilane, and C_3_H_8_O_3_ of glycerol

#### Identification of source analogues

The data gathered for the source analogues was mainly obtained from the SCCS report and the OECD WPMN dossier on nano-TiO_2_ [19, 24] (version published online in March 2016). The final dataset consisted of 6 TiO_2_ nanoforms with adequate data (see Table 1). The 6 nanoforms mainly vary in size (from 5 to 93 nm), coating (two of them are declared coated by the manufacturer and the others are declared without a coating), crystal type (anatase and rutile) and composition of the coating (hydrophobic or hydrophilic). NM- 100 is the largest of the NM with a primary particle diameter of size of 93 nm, anatase type, and uncoated. NM-101, instead is the smallest of the source analogues with a primary particle size diameter of 5 nm, of type anatase, declared uncoated and with a large amount of organic matter as impurities (8% w/w). NM-103 and NM-104 were very similar in size (24 nm), coating (both coated with Al_2_O_3_, dimethicone (C_2_H_6_OSi)_n_ and silane), and type (rutile). The main difference between them is the surface coating as NM-103 is hydrophobic (dimethoxydimethylsilane), while NM-104 is hydrophilic (glycerol). NM-102 has a particle size diameter of 22 nm, is uncoated, and of type anatase. NM-105 is also uncoated, has primary particle size of 20 nm, and is 83% anatase and 17% rutile.

### Step 2: Gather the available data for each group member and evaluate the data for adequacy and reliability; build a data matrix

The data collected for each source analogue can be found in Table SM4 and contains two clearly differentiated blocks of information: a) physicochemical characterisation, fundamental behaviour and reactivity; and b) toxicological data of the endpoint to read-across (comet assay in vitro genotoxicity).

The choice of properties to capture in the database was informed by the templates proposed by Schultz et al. [25], with adaptation to include specific NM properties [4, 14]. The properties collected corresponded to:

1. **What they are**: Name, JRC nanomaterials repository number, chemical composition, impurities, crystal type, crystal size, surface coating, porosity, basic morphology, primary particle diameter, average particle diameter, average length (TEM), aspect ratio, particle size distribution, pour density (weighing), specific surface area
2. **Where they go:** Agglomeration, dustiness, solubility(ies), dispersibility, (bio)persistence, redox potential, zeta potential, soelectric point, abiotic transformation, toxicokinetics,
3. **What they do**: Redox potential

#### Toxicological studies

A literature review on available genotoxicity studies was carried out. The references and corresponding reliability call assigned according to the ANSES criteria [21] can be found in Table SM5.

Table 2 shows the collected genotoxicity tests, specifically the comet in vitro tests of interest to this case study, in which the results are expressed as the number of positives out of the total number of (reliable) studies. The genotoxicity call for each source analogue was defined by the majority call with respect to the in vitro comet assays, i.e. a value of 1 was assigned when the majority of tests were positive, and 0 when the majority were negative. Results from bacterial mutagenicity test (Bacterial reverse mutation assay; Ames test) were not included in the count, as this test is not considered applicable to NMs in its current form [26, 27]. The in vitro micronucleus test is considered applicable to NMs after modification, and the in vitro comet assay is considered applicable to NMs [28, 29] but it is not a validated test in regulatory toxicology [30].

**Table 2:** Summary of genotoxicity results for the source NMs. The number of positives results over the total number of tests performed is indicated

	in vivo		in vitro		
	Micronucleus assay	Comet assay	Micronucleus assay	**Comet assay**	Genotoxicity (1/0)^a^
NM-100	–	–	–	**2/2**	1
NM-101	0/3	1/5	–	**2/6**	0
NM-102	0/6	2/13	3/10	**5/8**	1
NM-103	0/5	1/12	3/8	**0/6**	0
NM-104	0/5	2/12	3/8	**0/6**	0
NM-105	2/9	4/15	4/18	**10/14**	1

^a^ 1: NM is considered genotoxic in the in vitro comet assay; 0: NM is considered not genotoxic in the in vitro comet assay. The column highlighted in bold presents data used to determine the genotoxicity (1, 0) of each NM

#### Physicochemical parameters

The total non-TiO_2_ content of the source analogues varies from 0.11 to 11%, where the highest values are justified by the presence of coating. NM-103 and NM-104 contain 6% of Al_2_O_3_ and 2% of organic functionalisation (dimethicone, silanes, and dimethoxydimethylsilane for NM-103 making it hydrophobic; and tetramethyl silicate glycerol, silanes, hexadecanoic acid, methyl ester, octadecanoic acid for NM-104 making it hydrophilic) [20, 31]. NM-101 is a particular case in the sense that it was not declared as coated by the manufacturer [32], but which was found to have 9% of “organic impurities” consisting of silane, hexadecanoic acid, methyl ester, and octadecanoic acid [20]. This difference is reflected in Table 3 and Table SM4, where the presence of (declared) surface coating is represented by its % w/w and where the “Total non-TiO_2_” content accounts for the amount of matter that is not TiO_2_, thus including coating and impurities.

**Table 3:** Grouping hypothesis and read-across of comet assay results. TiO_2_ R and TiO_2_ A are the two target NMs. According to the grouping hypothesis based on the presence or absence of the coating, the two target NMs are assigned to the negative and positive group, respectively. Missing values are indicated with a dash (−)

Name		NM-100	NM-101	NM-102	NM-103	NM-104	NM-105	TiO_2_ R	TiO_2_ A
In vitro comet assay^b^		1	0	1	0	0	1	*0*	*1*
What they are	Total non-TiO_2_ content including coating and impurities (% w/w)	1.5	9	5	11	11	0.11	13	0.5
	Surface coating (%)	0	0	0	8	8	0	11	0
	Organic matter (% w/w)	0	8	0	2	2	0	9	0
	Crystal type (Anatase)	1	1	1	0	0	0.84	0	1
	Crystal type (Rutile)	0	0	0	1	1	0.16	1	0
	Crystal type (Cubic)	0	0	0	0	0	0	0	0
	Crystallite size (mean) (nm)	117.81	7.69	23.93	24.32	24.71	22.44	–	–
	Shape (elongated = 1, spherical = 0)	0	0	0	1	0	1	1	0
	Aspect ratio	1.53	1.53	1.53	1.7	1.53	1.36	6.2	1
	Primary particle diameter (mean) (nm)	93.45	5.25	22.00	24.00	24.50	20.13	62 × 10	14
	Specific surface area (m^2^/g)	9.23	316.07	77.87	53.98	54.33	47	177	149
Where they go	Isoelectric Point (Mean) (pH)	–	5.5	6	8.3	8.5	6.8	–	–
	Density (g/ml)	3.84	3.99	3.84	4.02	4.09	4.05	–	–
	Mean of total pore volume (ml/g)	0.032	0.319	0.300	0.262	0.194	0.194	–	–
	Micro surface area (m^2^/g)	0	13.625	1.108	0	0	0	–	–
	Micropore volume (ml/g)	0	0.00179	0.00034	0	0	0	–	–
	Dustiness-Respirable(mg/kg)	1500	5600	9200	19,000	6400	11,000	–	–
	Biodurability 24 h 0.05% BSA (Ti content) (μg/l)	5.2	0	0	0	0	0	–	–
	Biodurability 24 h Gambles solution (Ti content) (μg/l)	0	0	3388	0	0	0	–	–
	Biodurability 24 h Caco2 (Ti content) (μg/l)	796	3414	1741	222	3386	2724	–	–
What they do	Redox Caco2 medium^a^	1	-1	-1	1	-1	-1	–	–
	Redox Gamble’s solution^a^	1	0	-1	1	-1	-1	–	–
	Redox BSA^a^	0	0	0	0	0	0	–	–

^a^values obtained from deliverable 4.7 of Nanogenotox [33] determined by measuring the content of O_2_. Oxidising properties (1), neutral (0), reducing (−1)

^b^1: NM is considered genotoxic in the in vitro comet assay; 0: NM is considered not genotoxic in the in vitro comet assay. In vitro comet results are predicted for TiO_2_ R and TiO_2_ A (characters in italics in the last two columns)

The influence of the biological matrix on the particle size distribution of the NM is taken into consideration in our dataset by including NM particle size distribution, zeta potential and polydispersity index measured in different biological media (e.g. MilliQ water, Dulbecco’s modified eagle medium - DMEM - with and without L-glutamine, fetal bovine serum - FBS, and phosphate-buffered saline medium - PBS) and with different treatments (e.g. untreated, 1 min probe sonication, and 20 min ultrasound bath sonication). Solubility and redox potential are measured in Gamble’s solution (representing a lung fluid) and Caco2 medium (representing the intestinal environment). Inputs on solubility and biodurability were deducted by elemental analysis of the particle-free tested media [33]. For more information on the data analysis behind the values reported in Table SM4, please refer to section 1.2 in the Additional file 1.

#### Construct a matrix to identify available data

Table 1 summarises the information available on the source and target analogues in our case study, including also the genotoxicity based on the in vitro comet assay.

### Step 3: Grouping of nanoforms

#### Development of grouping hypothesis

The analysis of the literature and the data gathered in Table 3 yields the following grouping hypothesis:

*Nano-TiO*_*2*_ *in its uncoated form has the potential to damage DNA, but this can be masked by the presence of coating or large amounts of impurities on the surface of the NM.*

It can be readily seen in the dataset of analogues that the coated NMs turn out negative in the comet assay while the ones without coating and organic impurities turn out positive. This can be explained by both, direct genotoxicity or indirect primary genotoxicity [34]: The conduction band of TiO_2_ falls in the range of biological redox potentials [35], meaning that TiO_2_ with or without the presence of UV light can generate reactive species that react with cell constituents such as proteins or DNA. In both genotoxic mechanisms physical interaction between NM and DNA (i.e. direct) or another cellular component (e.g. enzyme mediated a redox reaction) that generates reactive oxygen species (ROS) (i.e. indirect) is necessary for the DNA damage to occur. The NM coating may act as a physical barrier that can prevent this contact between the surface of TiO_2_ and DNA or other cellular components [36]. Therefore following this rationale, coated nano-TiO_2_ will not turn out positive in the comet assay as there will be no physical interaction between the surface of the NM and DNA or cellular components. If NM aggregate/agglomerate, the deposition of NM in in vitro tests is higher. If the deposition is higher, the amount of NM and concentration seen by the cells is “de facto” higher than for an analogous situation with less deposition. Therefore, it seems evident that the effect of coating may in one way or another affect the outcome of an in vitro assay.

### Step 4: Assess the applicability of the approach and fill data gaps

#### Assess the grouping hypothesis

The applicability of the approach can be assessed by determining the robustness of the grouping hypothesis, i.e. assess the similarity within each group of NMs. Due to the lack of a uniquely defined structure, the similarity was defined by the physicochemical properties obtained for each nanoform in Table SM4. Different chemoinformatic techniques, two unsupervised and one supervised, were used to assess the grouping hypothesis.

##### Data reduction -

The initial dataset included 6 source analogues with approximately 147 properties for each of them (Table SM4). Two properties, crystal type cubic and redox activity in BSA, were discarded because their values were constant for all nanoforms. No correlation filter was applied to the dataset because the limited number of data points for each property (6 points) would overestimate the correlations and the filtering. However, some filtering was necessary because the dataset was biased towards Dynamic Light Scattering (DLS) measured properties, as it contained a total of 62 related properties that were measured in slightly different conditions, i.e. different media and treatments (see Step 2, physicochemical parameters). Consequently, the dataset had a high amount of particle size distribution, zeta potential, and polydispersibility index (PdI) measures. In order to reduce the weight of such measures and obtain a more balanced dataset, these properties were reduced to 4 measures each by using a hierarchical clustering of the transposed dataset (see section 1.2 of the Additional file 1). This allowed the determination of groups of similar properties from which one property for each set was randomly selected as representative of the rest.

##### Hierarchical clustering (HC) -

**Fig. 2:**
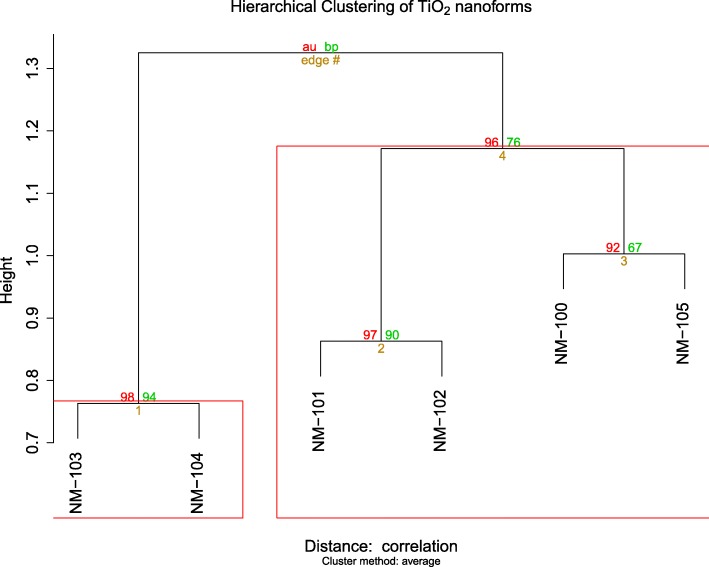
Hierarchical clustering of the TiO_2_ analogues. The numbers in red correspond to the “Approximately Unbiased” (AU) *p*-value that is computed by multiscale bootstrap resampling, and the ones in green to “Bootstrap Probability” *p*-value (BP), which is computed by normal bootstrap resampling. The height in the Y-axis indicates the distance between clusters computed as average linkage. AU *p*-value will be used for the interpretation as it is usually a better approximation to the real *p*-value

The HC of the obtained dataset, which contained 50 variables, is presented in Fig. 2 and shows that NM-103 and NM-104 form a very solid group (*p* < 0.01). The other 4 NMs form another group as they are clustered together [16] with high significance according to the approximately unbiased (AU) *p*-value that is computed by multiscale bootstrap resampling. It is worth mentioning that the clusters obtained here must be only considered from an exploratory point of view and in a weight of evidence context. This information alone cannot be used to define clusters of NMs but must be complemented with other techniques and rationales (e.g. PCA, variable selection, mechanistic information) to be used in read-across.

##### Principal component analysis (PCA) -

While the hierarchical clustering indicates possible groups of NMs by taking into account all physicochemical properties and forming subsequent groups of 2 substances, PCA is a dimensionality reduction technique that shows the properties that account for the maximum variance between individuals, i.e. the source NM in this case. PCA also uses all properties to determine each of the principal components (PC) but are weighted in such a way that a minimum number of properties can be used to explain the differences between the NMs.

**Fig. 3:**
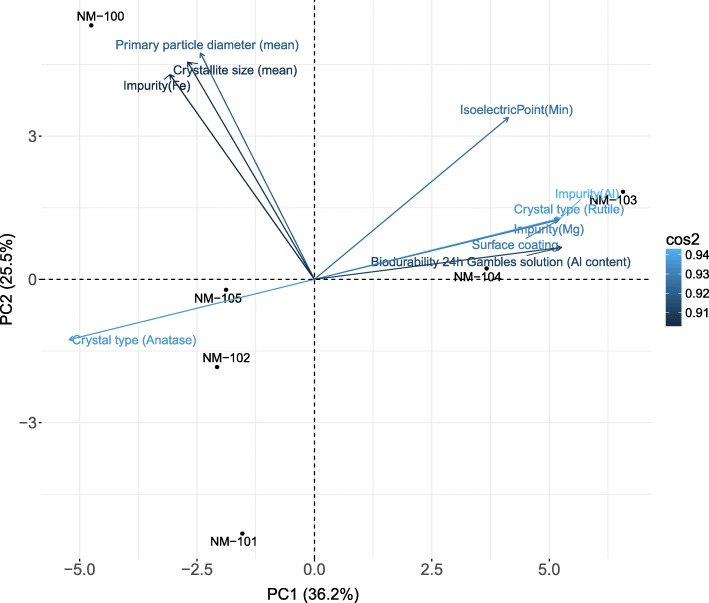
Principal component analysis (PCA) of the dataset of 6 TiO_2_ analogues. The position of the analogues (individuals) on the space of PC1 vs PC2 are indicated as black dots. Arrows correspond to the 10 variables with higher contribution to the PCs. The colours are defined by the squared loadings (cos^2^) and indicate their contributions to the PCs

PCA of the dataset of source analogues shows a similar picture to the one obtained in the HC (see Fig. 2). The NMs are placed in the plot by using the PC1 and PC2 scores. The loadings of each property with respect to PC1 and PC2 are indicated as arrows. NMs that appear close to each other indicate similarity in the space defined by PC1 and PC2. Long and light blue arrows indicate high contribution of that specific property to one of the PCs. The closer the arrow is to an axis, i.e. to a PC, the higher correlation with that PC. It is necessary to remember that PCA plots are simplifications of the whole picture and that the fact that NMs appear close to each other only indicates that these NMs are similar to each other in that reduced representation of reality given by 2 variables, i.e. PC1 vs PC2. PC1 and PC2 typically account for a rather large variance and their components indicate what the variables that differentiate NMs the most are. The fact that these variables be related with the endpoint of interest cannot be assured and is not the purpose of PCA or other unsupervised techniques. In Fig. 3, NM-103 and NM-104 appear close to each other at the positive side of PC2. The arrows show that these positions are mainly driven by the properties related to impurities of Al (Biodurability 24 h in Gambles solution - Al content), Mg, by the crystal type rutile, and % of surface coating. NM-100 appears at the top part of the plot mainly driven by particle primary diameter and crystallite size, which matches the fact that NM-100 is the biggest NM of the series (~ 93 nm, which can be considered as bulk material). For the same reason, NM-101 appears at the bottom of the plot as it is the smallest NM, and NM-102 and NM-105 appear next to each other on the negative side of PC1, mainly driven by crystal type anatase and by not having surface coating.

The squared loadings of the two first principal components are given in Table 4 and show that the properties with the higher contributions to PC1 are the biodurability 24 h Gambles solution (Al content) and impurity (Al), which are similar properties; crystal type (anatase and rutile), and % of surface coating and Mg impurity. For PC2 the main contributors are the specific surface area, total pore volume, primary particle diameter, crystallite size, and Fe impurities.

**Table 4:** Squared loadings of PC1 and PC2 of the PCA of the source analogues

Property	(PC1 loadings)^2^	Property	(PC2 loadings)^2^
Biodurability 24 h Gambles solution (Al content)	0.90	Specific surface area (mean)	0.77
Impurity(Al)	0.89	Mean of total pore volume (ml/g)	0.74
Crystal type (Rutile)	0.89	Primary particle diameter (mean)	0.73
Crystal type (Anatase)	0.89	Crystallite size (mean)	0.67
Surface coating	0.87	Micropore volume (ml/g)	0.63
Impurity(Mg)	0.87	Impurity(Fe)	0.63

The loadings also show that other properties like zeta potential, PdI, or particle size distribution have less influence.

##### Random forest variable selection -

The random forest variable selection algorithm is a supervised technique and uses the physicochemical properties to predict a given outcome, in this case positive or negative results in comet assays. It can provide a measure of relative importance of the variables for the prediction based on the times the variables were selected in the different trees. In this case, the Gini index was used as the target variable to optimise the trees [37].

**Fig. 4:**
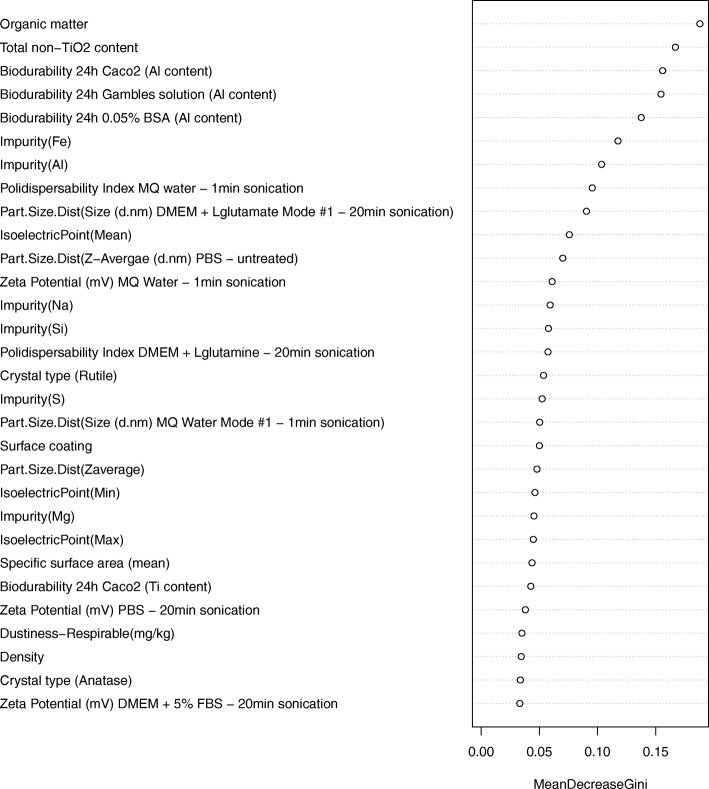
Relative importance of variables in terms of their predictivity of the comet assay. Variable importance expressed as mean decrease of the Gini index of the source nanoforms

The variable importance plot of the source analogues (Fig. 4) clearly shows that the most important variables to predict the comet assay results for the 6 analogues are the content of organic matter and total non-TiO_2_. The properties that follow in the list correspond to the biodurability measures (Al content) after 24 h of incubation in different media (Caco2, Gamble’s solution, and BSA). All these measures are directly or indirectly related to the presence of coating as the Al content and organic are mainly found on the coating.

#### Fill data gaps

HC, PCA and random forest variable selection algorithms supported the grouping hypothesis for the nano-TiO_2_genotoxicity tested with the in vitro comet assay.

The identification of the two target NMs in Table 3 includes the coating of the two nanoforms. According to the physicochemical properties of the identified target NMs, we can assume that they are included in the same variable space as the source NMs: primary particle size, shape, total non-TiO_2_ content, organic matter, crystal type, and specific surface area are included in the range of the source analogues. Because of the lack of some physicochemical data for the target NMs, it was not possible to include them in the PCA analysis or in the clustering exercise. However, it is possible to assign the two target NMs to a class according to some of their characteristics. Since the presence of coating or high amount of non-TiO_2_ content on the surface of nano-TiO_2_ appears to prevent NM to cause DNA damage, it is possible to group TiO_2_ R nano with the analogues NM-103, NM-104 and NM-101, which give negative results in the in vitro comet assay, and TiO_2_ A nano with NM-100, NM-102 and NM-105, which cause DNA damage. In fact, as shown in Table 1, TiO_2_ R has a coating, and thus it is predicted to have a negative outcome in the in vitro comet assay. TiO_2_ A, instead, has a relatively low level of impurities and no coating, for which we thus predict a positive result in the in vitro comet assay. The fact that TiO_2_ R has a rod-shape (62 × 10 nm) while the source analogues are rather spherical is not expected to influence the result. The aspect ratio is too small to consider that TiO_2_ R could cause an asbestos-like effect, and although the shape may influence the reactivity, it would still be masked by the coating which is the main driver of the toxic effect. The outcome of the read-across is confirmed by the in vitro comet assay carried out by Guichard et al. [23] which shows that TiO_2_ A is positive in the in vitro comet assay while TiO_2_ R is not.

#### Is the group robust enough?

DNA damage caused by nano-TiO_2_ may be classified as direct primary genotoxicity, indirect primary damage, or secondary genotoxicity [34, 38]. Direct genotoxicity assumes that DNA and NM are in contact [39]. Indirect primary genotoxicity may be elicited by interaction of NMs with nuclear proteins (involved in replication, transcription, and repair), disturbance of cell cycle checkpoint functions, ROS arising from the NM surface, release of toxic metal ions from the NM surface, ROS produced by cell components, and inhibition of antioxidant defence [40]. Finally, secondary genotoxicity may be elicited by ROS production in inflammatory cells via an inflammation signalling pathway [41, 42]. Most experimental studies point towards a mechanism of action for indirect primary genotoxicity via ROS [38], but other studies could not find a clear correlation between the level of ROS production and DNA damage (similar level of ROS at different concentrations of nanomaterials but increased DNA damage [43], or no correlation between amount of ·OH and ^1^O_2_ and DNA damage [44]).

Another relevant aspect in determining the validity of the grouping hypothesis is supporting evidence for the way in which the coating can prevent DNA damage, as the mode-of-action is not entirely clear. For instance, it was shown [45, 46] that the addition of PEG coating to nano-TiO_2_ increased the dispersion of NMs which resulted in lower cytotoxicity and genotoxicity. Magdolenova et al. [47] showed that the degree of dispersion of TiO_2_ NMs had an influence on the DNA damage in three cell lines. Agglomerates of less than 200 nm had no effect on genotoxicity while larger ones showed positive results. These results could be due to larger agglomerates precipitate and deposit on the cells increasing the actual exposure to the NM or even covering them completely and suffocating them. Another consideration is the effect that the use of media with proteins (e.g. BSA, FBS) can have on the results. If the NMs are surrounded by proteins, they are more dispersed, less prone to aggregation and deposition, and also less toxic as the “reactive” part is encapsulated (“hidden”) behind the protein corona. Another aspect that cannot be ignored when analysing the in vitro results of TiO_2_ is its photocatalytic activity, which can be even triggered by a simple fluorescent tube [48]. Thus, it is obvious that the mechanism of genotoxicity of TiO_2_ is not well defined and that there might be more than one that could even take place simultaneously. Probably the truth is the combination of all factors that have as common source the presence of coating either by preventing aggregation of NMs, deposition, and therefore reducing exposure, or by preventing physical contact with DNA and/or other cell components after uptake. However, what is relevant in this case is that the majority of studies agree with the hypothesis presented here which is the fact that coated nano-TiO_2_ show fewer positive results in the in vitro comet assay than the uncoated ones, therefore it can be fairly concluded that the presence of coating reduces the genotoxic effects of nano-TiO_2_. It is important to keep in mind that the present coatings are mainly not “charged” as could be coatings with reactive or non-neutral groups such as terminal –COOH or –NH_2_, in which cases the grouping hypothesis might change.

#### Uncertainty evaluation

The AEs of the RAAF scenario 6 were used to systematically identify uncertainties in the grouping and read-across process. Uncertainties related to some aspects of the case study are discussed in more detail below.

Table 5 provides a summary structured according to the RAAF AEs, and also highlights the nanospecific considerations to be taken into account when applying the RAAF to NMs. Overall, the uncertainties were related to the i) complexity of nanostructures, affecting the definition of similarity and category boundaries; ii) nanomaterial identification and physicochemical characterisation, due to high measurement variability; iii) a limited dataset, iv) quality and inconsistency as well as reproducibility of study data due to missing SOPs protocols or uncertainty in their applicability to nanomaterials; v) finding correlations and identifying the physicochemical properties driving the toxicity; vi) limited knowledge about the mechanism of action (MoA).

**Table 5:** Evaluation of the uncertainties of the TiO_2_ read-across case study according to the ECHA RAAF scenario 6

	RAAF Assessment Element (Scenario 6)	Uncertainties in the TiO_2_ case study	Nanospecific issues
C.1	Substance characterisation	• Measured physicochemical characteristics of the NMs vary: measurement uncertainty. Is there an influence on other properties of the nanomaterials? • Impurity information not always available or inconsistent	• Physicochemical characterisation of NMs: high variability of measurements (influence of different experimental conditions)
C.2	Structural similarity and category hypothesis	• NM-101 is not declared as coated, but has 9% organic impurities that could be considered as a coating. • Different composition of the coatings/impurities (e.g. some containing Al_2_O_3_, dimethoxydimethylsilane, or glycerol) • Uncertainty of reading across a spherical particle to a rod-shaped particle	• For NMs, the similarity cannot be based on chemical (e.g. molecular) structure as for conventional chemicals, but should consider physical form and key physicochemical properties
C.3	Link of structural similarities and structural differences with the proposed property	• Little is known about the mechanisms of toxic action, making it challenging to link similarity to the endpoint (genotoxicity) considered	
C.4	Consistency of effects in the data matrix	• Uncertainty in applying existing testing protocols to nanomaterials and thus uncertainty in assessment of quality, reliability and relevance to human health endpoints of measured toxicity data	• Artefacts affecting the results of toxicity assessment of NMs are discussed in the literature
C.5	Reliability and adequacy of the source study(ies)		
C.6	Bias that influences the prediction	• Selection of analogues based only on data availability	
6.1	Compounds the test organism is exposed to	• The mechanism of genotoxicity of TiO_2_ is not well defined. It is also possible that several effects take place at the same time.	• For conventional chemicals, either the parent molecule or (bio)transformation products are the indirect/direct toxicants; for NMs the considerations extend to coating, released metals etc.
6.2	Common underlying mechanism, qualitative aspects		
6.3	Common underlying mechanism, quantitative aspects		
6.5	Occurrence of other effects than covered by the hypothesis and justification		
6.4	Exposure to other compounds than to those linked to the prediction	• For example the presence of reactive transition metals may also contribute to oxidative DNA damage induction.	

## Discussion

Nano-TiO_2_ was selected as case study because of its importance in the market [49], data availability [9, 19, 24, 50, 51], and in-house experience from related projects (ENPRA, NanoMILE, NanoTEST, ENRHES).

A simplified version of the workflow proposed by ECHA [4] for the read-across of NMs was applied in this manuscript (see Fig. 1). This simplified workflow collects all the available data in the first steps and avoids the generation of grouping hypothesis with insufficient data.

The read-across was documented by providing mechanistic interpretation of the available data, where possible, and according to the state of the art in the field. Chemoinformatic techniques such as HC, PCA, and random forest variable selection were used to support the grouping hypothesis of NMs.

Genotoxicity of TiO_2_ nanoforms as determined by in vitro comet assay was selected as endpoint to read-across. Although nano-TiO_2_ are well studied and data rich NMs, only 6 NMs with full data could be gathered. In vitro comet assay was deemed as the more suitable/relevant endpoint for the read-across case study, unlike the other endpoints, it provided two groups of NMs (genotoxic vs non-genotoxic) and a relatively high amount and diverse set of NMs.

Different issues arise when trying to read-across NMs with data collected from different sources. Data quality and variability are significant challenges in the field of nanotechnology [52]. As it is reported in the next paragraph, identification of nanoforms can be controversial [53, 54] as in the nano-TiO_2_ case different amounts of impurities and different sizes are reported for the same target substance and this contributes to increase uncertainty on the first step of the grouping for read-across procedure, consisting of the NM identification. Furthermore, the fact that the mode-of-action of nano-TiO_2_ genotoxicity is not (yet) well understood [55] complicates the formulation and assessment of grouping hypothesis, the basis of read-across. The necessary modifications to adapt the RAAF [22] to the read-across of nanomaterials were identified, and this is a key step to increase the use and certainty when reading-across nanomaterials.

The issues mentioned above together with the lessons learnt are discussed next.

### Data variability

Data variability in the reported parameters was mainly due to the lack of SOPs that leads to the application of different tools or approaches in the measurement of the same property (e.g. crystallite size). In the particular case of NM-100, four different values were collected: 141, 61, 168, and 100 nm. In order to transform ranges of values into single values suitable for read-across analysis, some data treatment was necessary. In general, if the distribution of values is normal, the mean values are a good representation of the reality, but if the distribution is not normal and there are extremes, then the median is a better option. For some parameters (e.g. primary particle size) the variability was rather low and, therefore where possible, it was decided to use the average values. In cases in which different techniques with varying precision provided significantly different results (e.g. specific surface area determined by BET or SAXS), the values provided by the most precise techniques were preferred (see section 1.2 of the Additional file 1 for further details on the data treatment).

The variability in the measurements can be misleading for the characterisation of nanoforms and thus in identifying similar analogues. For example, the physicochemical properties of the target substances showed that the measured ones were slightly different from those reported by the manufacturer. Guichard et al. [23] found for TiO_2_ R nano 11% w/w of impurities corresponding mainly to SiO_2_ (manufacturer declared up to 0.5%), the measured particle size corresponded to a rod of 62 × 10 nm (manufacturer declared 40 × 10 nm), and the surface area to 177 m^2^/g (manufacturer declared 50 m^2^/g). For the purpose of this study it was assumed that the substance tested in Guichard et al. corresponded to a coated TiO_2_ manufactured by Sigma. It is not clear though where is the limit to consider that two substances are the same.

### Determining similar NMs

One of the challenges of the case study was the identification of similar analogues as it had to be based on the physicochemical properties. The task was rather easy for some of the properties. For instance, NM-102, NM-103, NM-104, and NM-105 had particle diameter (TEM) of 22 ± 10 nm, specific surface areas between 77 and 47 m^2^/g, crystal types of rutile, anatase or combination of both (83% anatase 17% rutile for the case of NM-105). However, it resulted highly complex for properties such as particle size distribution (see Annex IX in Worth et al. [56]) or impurities.

The case of impurities was unexpectedly challenging. Impurities are defined as “an unintended constituent present in a substance as manufactured” [57], while surface coating consists in the surface chemistry purposely added to the NM. The measurement of the elements present on the surface of the NM does not distinguish between the two. In the present case, NM-103 and NM-104 were declared coated and were found to contain 6% of Al_2_O_3_ and 2% of organic functionalisation (dimethicone, silanes and dimethoxydimethylsilane for NM-103 making it hydrophobic; tetramethyl silicate, glycerol, silanes, hexadecanoic acid, methyl ester, octadecanoic acid for NM-104 making it hydrophilic). NM-101 was not declared coated but it was found to contain a high amount of impurities accounting for around 9% of the total weight. The composition of these impurities (silane, hexadecanoic acid, methyl ester, and octadecanoic acid) was very similar to the coating of the other NMs. In fact, the Nanogenotox project considered them as coating [32], but it was not deemed appropriate in this work as it would contradict the definition of impurities [57]. Since it was impossible to determine whether these impurities were added on purpose and in order to reflect its presence, we defined a new property named “Total non-TiO_2_ content including coating and impurities (% w/w)” which corresponded to the sum of all materials that were detected in the NM other than the core material, thus going beyond the surface coating declared by the manufacturer. This measure included also the coating, which was separately declared by the supplier and was also reported separately in our dataset as “Surface chemistry (as declared by manufacturer)” and “Surface coating (%)” indicating the quantity of coating with respect to the total weight of the NM. This way, 2 groups of NMs could be clearly identified, those with a high amount of non-TiO_2_ content (> 9% w/w), and those with lower or no amount of non-TiO_2_ content (≤ 5% w/w).

### Validity of the grouping hypothesis

Chemoinformatic tools such as HC and PCA can be used to process and extract knowledge from large amounts of data. We applied HC, PCA, and a variable selection algorithm based on random forest to support the grouping hypothesis of the read-across exercise.

HC and PCA of the source analogues showed that two groups of NMs can be clearly defined based on their physicochemical properties (see Fig. 2 and Fig. 3). HC can be used to determine similar NM with respect to their properties without biasing the similarity or weighting any of the properties. Following this principle, HC showed that NM-103 and NM-104 (negative in the in vitro comet assay) formed a very strong group (*p* < 0.01). In fact, both NMs were almost identical, of rutile type with a size of ~ 24 nm, and coated. The “only” difference was on the surface chemistry, which in one case was hydrophobic, and in the other hydrophilic. Thus, the analysis of the HC results shows that NMs are clustered according to crystal type, size and presence of coating.

Unlike HC, the PCA can show clusters of similar NMs as well as the properties that define their (dis)similarity. The properties that contribute the most to the PC are those that determine the main differences between the groups of NMs. The main contributors to the PCs were mainly related to the presence of impurities, biodurability, coating, crystal type (anatase vs rutile), particle size, and pore volume (see Table 4). The fact that crystal type variables appeared so high in the list is partially due to the values used to code each crystal type. Since most of the particles were either 100% anatase or 100% rutile, the differences between the anatase and rutile NMs (100% vs 0%) were highly significant. Primary particle diameter was also found to be one of the main differences between NMs as the biggest one was 93 nm and the smallest 5 nm. Biodurability 24 h Gambles solution (Al content) and impurity(Al), both highly related to coating, are very similar properties as the former one corresponds to the quantity of Al dissolved in media after 24 h, and the second one corresponds to the quantity of Al found after calcination of the NMs.

The PCA showed a cluster formed by NM-102 and NM-105. Both are positive in the comet assay and both correspond to uncoated anatase TiO_2_ (100% and 84%, respectively) with ~ 23 nm and low amount of impurities. NM-100 does not cluster together with any of the other NMs in the PCA because it corresponds to a relatively large “NM” (~ 98 nm), which makes it significantly different from the rest. In fact, PC2 has a strong component of particle size what pushes NM-100 at the higher part of the plot. However, if only the crystal type and coating were considered, NM-100 would group with NM-102 and NM-105 as it is uncoated, and 100% anatase. Such a classification would match the toxicological profile of these NMs as they all turn out positive in the comet assay. However, this classification would not hold for the other NMs, as NM-101 is also anatase but negative in the comet assay. As mentioned above, NM-101 is a complex case and it is difficult to classify. It is the smallest of all NMs with a diameter of 5 nm (lower part of the PCA), it is of anatase type, and although it is declared uncoated by the producer, it contains a high amount of impurities (9%), which are of similar composition to the coating of NM-103 and NM-104. The results from PCA show that the NMs differences are mainly driven by presence of impurities, biodurability, coating, crystal type, particle size, and pore volume.

Finally, the random forest analysis supports our grouping hypothesis. The variable importance plot in Fig. 5 shows that the properties organic matter and Total non-TiO_2_ content are the most discriminating properties to predict in vitro comet assay results. Both properties are related to the presence of coating or impurities, thus, it is clear that there is a correlation between the NMs that have coating and/or organic impurities and the result of the in vitro comet assay. The fact that the presence of coating and/or organic impurities can explain the result of the in vitro assay does not imply that they are the only ones that are relevant. In fact, the chemoinformatic techniques have shown several properties that account for the similarity and clustering of these NMs and that may also be important to understand the outcome of the in vitro comet assay.

One valuable question is what would be the outcome for a NM of type rutile and uncoated. We do not dispose of such a NM in the group of source analogues, therefore, such a read-across would be more uncertain than the current one. Following the present grouping hypothesis, uncoated rutile would also be predicted as positive in the in vitro comet assay because the grouping hypothesis is based on the presence of coating. It would be desirable to dispose of data for this type of nanoform before performing such a read-across so as to have a prediction with less uncertainty.

### Uncertainty evaluation according to the ECHA RAAF

The case study shows that the RAAF is applicable to NMs. A few nanospecific issues were identified which should be accommodated when applying the RAAF to NMs. First of all, the consideration of similarity should be extended from being based on the chemical structural to other appropriate parameters such as the physical form and key physicochemical properties. Additional sources of uncertainty to be considered for NMs are the high variability of measurements for NM characterisation as well as the uncertainty of adequate application of testing protocols to NMs, including possible NM-specific artefacts, and thus uncertainty of reliability and relevance of toxicity assay data. In the RAAF scenarios, the toxicant is either the parent chemical or a biotransformation product, for NM additional possibilities might be considered, including for example the coating or release of metals. For defining identical or different compounds – as basis for differentiating RAAF scenarios – factors such as surface coating and size should also be considered. With the knowledge on NMs further increasing in the future, possible identified NM-specific mechanisms of toxicity should also be taken into account.

## Conclusions

In this work, we successfully applied a simplified version of the workflow for grouping and read-across proposed by ECHA [4] to read-across nanoforms of TiO_2_. We collected and curated all public information available for nano-TiO_2_. In vitro comet assay was selected as the endpoint to read-across as it turned out to be the endpoint with the largest number of NMs that could be assigned to either a positive or negative outcome. The final dataset that was used for the read-across was composed of 6 nano-TiO_2_ with more than 100 physicochemical properties. Two groups of nano-TiO_2_ were identified based on their physicochemical properties. A grouping hypothesis that reads: “*Nano-TiO*_*2*_*in its uncoated form has the potential to damage DNA, but this can be masked by the presence of coating or by the large amounts of impurities on the surface of the NM*” was used to successfully read-across the in vitro comet assay results of two nano-TiO_2_. In order to extend this hypothesis to be able to determine whether nano-TiO_2_ is genotoxic, it would be necessary to repeat the exercise considering other genotoxicity tests, as the in vitro comet assay has been shown to be prone to give false positives [58].

It was shown how chemoinformatic techniques such as HC, PCA, and random forest may be used to support or evaluate a grouping hypothesis by determining (dis)similar NMs as well as the properties that differentiate them the most. Furthermore, it was shown that the ECHA RAAF to evaluate the confidence in a read-across argument is also applicable to NMs provided some modifications are made in order to take into consideration NM specificities such as the extension of the basis for similarity beyond chemical structure.

The main challenges that were faced during the read-across exercise were: i) identification of the (non-)nanoforms, ii) experimental variability associated with the physicochemical and toxicological information, iii) lacking measurement protocols, iv) the lack of knowledge on the mechanisms of genotoxic action of NMs. Current efforts in the scientific community are ongoing to address knowledge gaps and availability of SOPs [59–61]. These developments will support nanosafety assessments, including the development of read-across case studies.

## Additional file

Additional file 1: Grouping of nanomaterials to read-across hazard endpoints: from data collection to assessment of the grouping hypothesis by application of chemoinformatic techniques. (DOCX 1335 kb)

## Footnotes

1 In the context of this paper, a nanoform is a form of a substance which fulfils the EC recommended definition of nanomaterial and is characterised also by shape and surface chemistry [62].
