# Supplementary material for: Grouping of nanomaterials to read-across hazard endpoints: from data collection to assessment of the grouping hypothesis by application of chemoinformatic techniques
Source: Part Fibre Toxicol. 2018 Sep 24;15:37. doi: 10.1186/s12989-018-0273-1 (PMC6154922; doi:10.1186/s12989-018-0273-1)
Supplement: Supplementary file 1 — Grouping of nanomaterials to read-across hazard endpoints: from data collection to assessment of the grouping hypothesis by application of chemoinformatic techniques. (DOCX 1335 kb) [file 12989_2018_273_MOESM1_ESM.docx]

**Additional File**

**Grouping of nanomaterials to read-across hazard endpoints: from data collection to assessment of the grouping hypothesis by application of chemoinformatic techniques**

L. Lamon,^a^ D. Asturiol,^a, *^ A. Richarz, E. Joossens, R. Graepel, K. Aschberger and A. Worth

a: Equal contribution

* Asturiol, D.; Directorate General Joint Research Centre, Directorate F – Health, Consumers and Reference Materials, Chemicals Safety and Alternative Methods, Ispra (VA) Italy; email: [david.asturiol-bofill@ec.europa.eu](mailto:david.asturiol-bofill@ec.europa.eu)

Contents

[1. Data Treatment 2](#_Toc496613396)

[1.1. Compilation of the initial dataset 2](#_Toc496613397)

[1.2. Reducing the initial dataset to a single row of data 3](#_Toc496613398)

[Impurities 3](#_Toc496613399)

[Crystal type 3](#_Toc496613400)

[Crystallite size 3](#_Toc496613401)

[Surface chemistry 4](#_Toc496613402)

[Primary particle diameter 5](#_Toc496613403)

[Particle size distribution 5](#_Toc496613404)

[Zeta Potential 7](#_Toc496613405)

[Polydispersibility Index (PdI) 8](#_Toc496613406)

[Isoelectric point 9](#_Toc496613407)

[Density 10](#_Toc496613408)

[Porosity 10](#_Toc496613409)

[Specific surface area 10](#_Toc496613410)

[Dustiness (respirable) 10](#_Toc496613411)

[Biodurability 10](#_Toc496613412)

[Redox potential 10](#_Toc496613413)

[1.3. Full data matrix 11](#_Toc496613414)

[2. Literature search on genotoxicity studies 15](#_Toc496613415)

[2.1. List of the genotoxicity studies taken into consideration 17](#_Toc496613416)

## Data Treatment

## Compilation of the initial dataset

In order to compile all the available data for the case study a dataset for each nanoform (NM-100 –> NM-105) was created. The data was gathered in an excel file with columns defining properties such as impurities (Al, Si, P, etc.), crystal type, crystal size, surface coating, primary particle diameter, particle size distribution, etc., and rows corresponding to different entries of data.


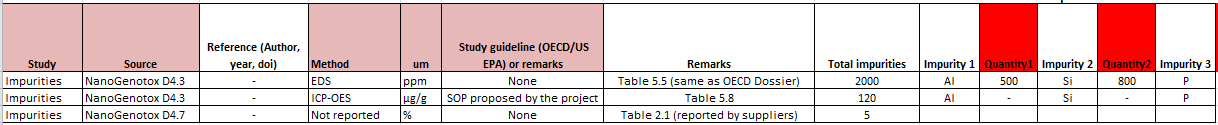


**Figure SM1.** Example of the dataset created for NM-102. The first columns (pink) correspond to metadata and linkers to the data source, and the rest of columns to the data obtained.

The columns of the dataset were defined on-the-fly as the data was being processed, and the data type was defined by the type of data that was available in the sources. For instance, since the particle size diameter section was obtained from SAXS/USAXS data, the following columns were registered:

- Mean particle diameter (nm)
- Gyration radius of primaries (nm)
- Gyration radius of aggregates(nm)
- Fractal dimension
- Number particles/aggregate
- Aggregated diameter

Similarly, the particle size distribution section contained columns for the measures in each media, e.g. PBS, DMEM + X% FBS, MQ Water, etc.

Each row of the dataset corresponded to data obtained from one test. For instance, all impurities measured with EDS were inputted in the first row under the corresponding columns. Each new source of data was inputted as a new row. In case the same type of data was found in a different source, the data was inputted in a new row but under the same column that was created previously. In order to keep track of the sources of the data, the first columns of the dataset were dedicated to store metadata (see Figure SM1) such as the test method (e.g. EDS, DLS, XRD, BET), source of data (e.g. Nanogenotox deliverable D4.3), units of the data (e.g. nm, %, ppm, µg/g), guideline used, or remarks.

This task yielded a dataset that was mostly empty as each new entry added a new row but only the property of interest was filled with data and the rest are left blank. The initial dataset of NM-102 was comprised of 7260 cells (44 rows x 165 columns), 464 of which contained some data, i.e. 6%.

## Reducing the initial dataset to a single row of data

The next step to obtain a dataset for read-across that could be used, consisted of merging the data available in the columns into a row of data, which means that each column was reduced to a single value.

### Impurities

Data on impurities was obtained from two different instruments, EDS and ICP-OES. In most cases, both instruments detected different atoms and there was no need to merge the data. However, for some cases like Impurity of K of NM-100 two values were present, 2500 ppm (EDS) and 1000 µg/g (ICP-OES). In this case, the different precision of the instruments was not considered. Instead, the precautionary principle was applied and only the largest value was considered, i.e. 2500 ppm.

The same strategy was used for all impurities.

### Crystal type

This data corresponded to the percentage of crystal. The decision for most of the NMs was not complicated as they were measured as 100% anatase or 100% rutile. Only for NM-105 two measures from different labs using the same technique were obtained, one with 86% anatase and the other one 81% anatase. It was decided to average the two values giving a final value of 83.68% of anatase and 16.32% of rutile.

### Crystallite size

Crystallite size is one of the problematic measures because crystallite sizes are measured with XRD but different algorithms can be used to determine the sizes (see Nanogenotox 4.3 pag. 14 for further information), e.g. Scherrer equation, Peak fit FWHM, Topas, Fullprof. In addition, measures from three different labs were obtained for this property.

The crystallite size measures for the small NMs, i.e. NM-101 - NM-105 showed a rather low variability independently of the laboratory or methods used to determine it. But some cases showed unexpected differences: For instance the laboratories IMC-BAS and NRCWE used the same algorithms and instrument to measure the “same” NMs, but the measures obtained by NRCWE were systematically larger than those of IMC-BAS, ~25% larger. The case of NM-102 is significant as although the larger size difference is only of 12nm, it is with respect to a measure of 18-30nm, which represents more than 50% variation. The authors acknowledge these differences and state that most of them can be explained by the differences of instrumental performance, which has an estimated standard deviation of 5 nm.

Different is the case of NM-100, whose values were rather variable (from 57-168 nm). The reason for this is probably the fact that XRD is not adequate to measure crystals of more than 100 nm as the authors stated in the report. In addition, the producers indicate a size of 200-220 nm, but one of the measures determined a crystal size of 57 nm. Therefore, it was difficult to determine what value was the right one. Like in most cases, the truth probably was somewhere in between the extremes. It is worth mentioning that NM-100 corresponded to a dry milled NM what could explain the presence of particles of different sizes.

When dealing with samples with large variability or having extreme values, it is usually advisable to use the median of these values. The median of the measures obtained for NM-100 was 120 nm. However, since the average was 117 nm and the average was used for the rest of NMs, it was considered appropriate to use for NM-100 the average value of 117 nm.

### Surface chemistry

Surface chemistry is per se a difficult category because of the “legal” definition of surface chemistry and coating and the tests that are used to determine it. Surface chemistry and coating are defined as substances that are intentionally added to the surface of the nanomaterial and therefore it depends on the declaration of the manufacturer. NM surface chemistry is usually determined by the w/w percentage of each of the constituents of the NM. The method used to determine the surface chemistry of NMs is usually Thermogravimetric analysis, which consists of the monitoring of the weight of the sample while increasing the temperature up to 1000 ºC. The thermogravimetric analysis is usually complemented with an elemental composition analysis that is carried out after calcination. As it stands, the thermogravimetric analysis cannot distinguish between substances that were intentionally added to the surface and those that were not intentionally added.

In the current case study, 2 NMs (NM-103 and NM-104) were declared as coated with Al_2_O_3_ and silicon dimethicone, and the rest of NMs were declared uncoated by the manufacturers.

As expected, the thermogravimetric analysis of NM-103 and NM-104 showed a mass loss that matched the declaration of coating by the producer, but NM-101 showed an unexpected similar mass loss (~8%) at the same temperature and of the same composition as NM-103 and NM-104. Since the raising of the temperature calcinates both surface coating and impurities, it is impossible to know if the mass loss of NM-101 corresponded to impurities or to undeclared coating. Therefore, since NM-101 was declared uncoated, the mass loss cannot be considered as surface chemistry but has to be considered as impurities.

In order to indicate this difference, a row consisting of “organic matter” impurities was added to the data matrix (see Table SM1). Similarly, a row named “Total non-TiO_2_ content including coating and impurities (%w/w)”, which corresponded to the sum of surface coating and impurities, was also added to the matrix.

**Table SM1.** Data used in the grouping hypothesis and read-across of comet assay results of nano-TiO_2_.

| ***Name*** | **NM-100** | **NM-101** | **NM-102** | **NM-103** | **NM-104** | **NM-105** |
| --- | --- | --- | --- | --- | --- | --- |
| ***In vitro* comet assay** | **1** | **0** | **1** | **0** | **0** | **1** |
| **Total non-TiO_2_ content including coating and impurities (% w/w)** | 1.5 | 9 | 5 | 11 | 11 | 0.11 |
| **Surface coating (declared) (%)** | 0 | 0 | 0 | 8 | 8 | 0 |
| **Organic matter (%)** | 0 | 8 | 0 | 2 | 2 | 0 |
| **Impurity(% w/w Fe)** | 0.49 | 0 | 0.07 | 0.06 | 0 | 0.06 |
| **Impurity(% w/w Si)** | 0.28 | 0.29 | 0.08 | 0.68 | 0.018 | 0.07 |
| **Impurity(% w/w K)** | 0.25 | 0 | 0.001 | 0.001 | 0.001 | 0 |
| **Impurity(% w/w P)** | 0.21 | 0.27 | 0.001 | 0 | 0 | 0 |
| **Impurity – coating (% w/w Al)** | 0.09 | 0.09 | 0.05 | 3.4 | 3.2 | 0.04 |
| **Impurity(% w/w Cr)** | 0.03 | 0 | 0 | 0 | 0 | 0 |
| **Impurity(% w/w Zr)** | 0.005 | 0.01 | 0.005 | 0.001 | 0.001 | 0 |
| **Impurity(% w/w Ca)** | 0.001 | 0 | 0.005 | 0.005 | 0.01 | 0 |
| **Impurity(% w/w Na)** | 0.001 | 0.1 | 0.001 | 0.01 | 0 | 0.001 |
| **Impurity(% w/w S)** | 0 | 0.22 | 0.001 | 0.01 | 0.01 | 0.26 |
| **Impurity(% w/w Mg)** | 0 | 0 | 0 | 0.001 | 0.001 | 0 |

### Primary particle diameter

Data from different sources and methods were obtained for the primary particle diameter. The most common technique to determine the primary particle size is TEM. In case different values from TEM were obtained, the average value was considered. In the present case study, however, data from SAXS/USAXS corresponding to the radius of gyration of primary particles was also found. But since data for NM-100 and NM-101 were missing, it was decided not to consider the data from SAXS/USAXS due to data gaps and keep only the TEM related data as data was available for all NMs.

### Particle size distribution

Data on the particle size distribution and aggregation/agglomeration was gathered for the NMs from: a) different sources, b) different methods (DLS and PCS), c) different media (e.g. MQ Water, PBS, DMEM + 1-10% FBS, DMEM + L-Glutamine), d) different treatments (unsonicated, 1min probe-sonication, 20min bath sonication). In addition, different measures (e.g. Zeta-size, Intensity distribution main peak, and FWHM main peak) were obtained for some of the NMs, only.

Since NM-100 and NM-101 were missing data for the Intensity distribution main peak and FWHM main peak, these parameters were not considered in the analysis.

#### Data in different media and treatment

Data for DLS measured as Z average was averaged between the different sources. Instead, the data obtained from PCS in different solvents and treatments was treated individually. Table SM2 contains different measures of particle size distribution with different solvents and treatments. Additionally, the data shows that measures such as the untreated sample in MQ water are bimodal as it contains sizes of 1115nm and 5170nm. In some cases, 3 modes were detected. This problem is reproduced in the corresponding measures of PdI and Zeta Potential. The differences of particle sizes indicate that the NMs agglomerate in a different way depending on the media and sonication treatment. In general they cannot be averaged between them unless a common behavior is observed. For instance, NM-102 shows very similar sizes in DMEM + 1,5,10% FBS as they correspond to 1415, 1414 and 1521 nm. However, NM-101 shows a bimodal of 1201/5232 for DMEM + 1% FBS, 1272 and 1406 for DMEM + 5 and 10% FBS, respectively. This shows that it is impossible to use a common merging technique and that the data needs to be treated individually.

An additional problem is the fact that not all media and treatments show bimodal or trimodal distributions. In order for the computer to be able to process the data, it was decided to include a row of data for each mode. In order to avoid blanks for the modes 2 and 3 of the monomodal cases, the value obtained was copied to mode 2 and mode 3. An example of the final data matrix is shown in Table SM2

**Table SM2.** Example of particle size distribution data in different media and treatments. Values in red indicate that the distribution was monomodal and that the same value was used for mode 1 and mode 2.

| ***Name*** | **NM-100** | **NM-101** | **NM-102** | **NM-103** | **NM-104** | **NM-105** |
| --- | --- | --- | --- | --- | --- | --- |
| **Particle size distribution in MQ Water, untreated, Mode #1 (nm)** | 391.2 | 1609 | 115 | 973.2 | 727.8 | 1102 |
| **Particle size distribution in MQ Water, untreated, Mode #2 (nm)** | 4862 | 1609 | 5170 | 973.2 | 727.8 | 204.7 |
| **Particle size distribution in PBS, untreated, Mode #1 (nm)** | 1440 | 1188 | 1528 | 1977 | 1817 | 4526 |
| **Particle size distribution in PBS, untreated, Mode #2 (nm)** | 5236 | 5148 | 5330 | 1977 | 5194 | 1150 |
| **Particle size distribution in DMEM + Lglutamate, untreated, Mode #1 (nm)** | 995.5 | 1438 | 2745 | 2255 | 3059 | 1881 |
| **Particle size distribution in DMEM + Lglutamate, untreated, Mode #2 (nm)** | 995.5 | 5560 | 2745 | 2255 | 3059 | 5372 |
| **Particle size distribution in DMEM + 1% FBS, untreated, Mode #1 (nm)** | 736 | 1201 | 1415 | 1040 | 1156 | 2454 |
| **Particle size distribution in DMEM + 1% FBS, untreated, Mode #2 (nm)** | 736 | 5232 | 1415 | 4593 | 5211 | 626.5 |

This strategy allowed the inclusion of all data but rendered a dataset with a number of particle size distribution values (54 for each NM). It is obvious that having 54 values for the particle size distribution of a NM is unnecessary and does not provide relevant information. In addition, it highly biases the dataset towards particle size distribution as it corresponds to more than 1/3 of the total number of physicochemical variables. In order to reduce the dimensionality of the particle size distribution part of the dataset, hierarchical clustering of the transposed dataset was used to find “similar” combinations of media-treatments. Figure SM2 shows the resulting clustering with the values that were randomly selected from each cluster.

Such a dimensionality reduction is not expected to be representative of the original data or an example to follow to reduce the dimensionality. Since it was deemed necessary to reduce the weight of the particle size distribution part of dataset and there was no apparent rational, the solution of the hierarchical clustering and random selection was chosen as an objective way of doing it. The use of the hierarchical clustering assured that the measures chosen did not show similar behavior and contributed to capture differences between NMs.


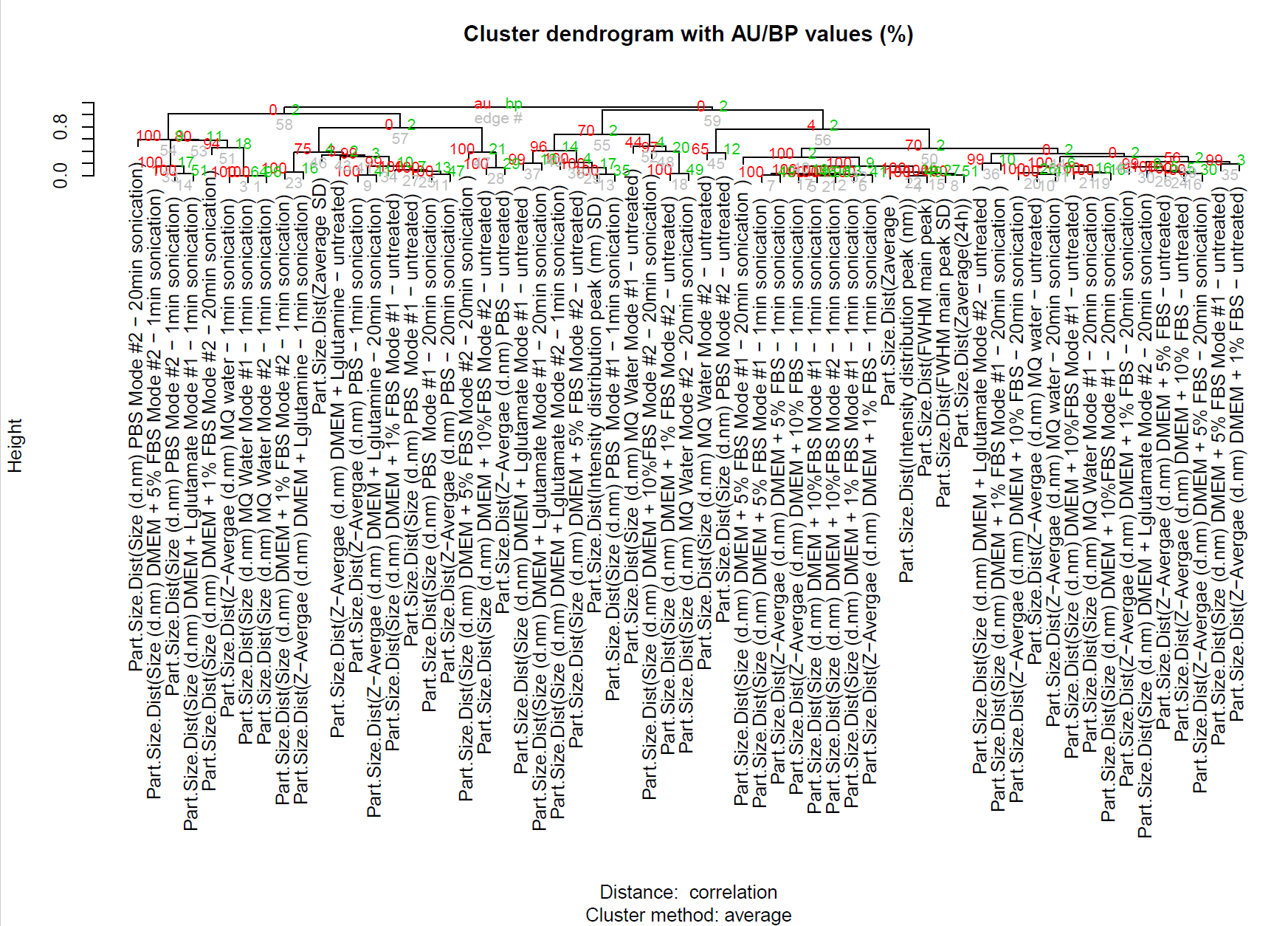


**Figure SM2.** Hierarchical clustering of the transposed matrix of particle size distribution measures obtained for the nano-TiO_2_. Arrows indicate the medium-treatment properties that were randomly selected from each cluster as representative.

### Zeta Potential

Since the Zeta Potential data is obtained with the same instrument and test as the particle size distribution and PdI, the reader is referred to the particle size distribution section for further details. The hierarchical clustering of the transposed matrix of Zeta Potential with the randomly selected properties for each cluster is shown below.


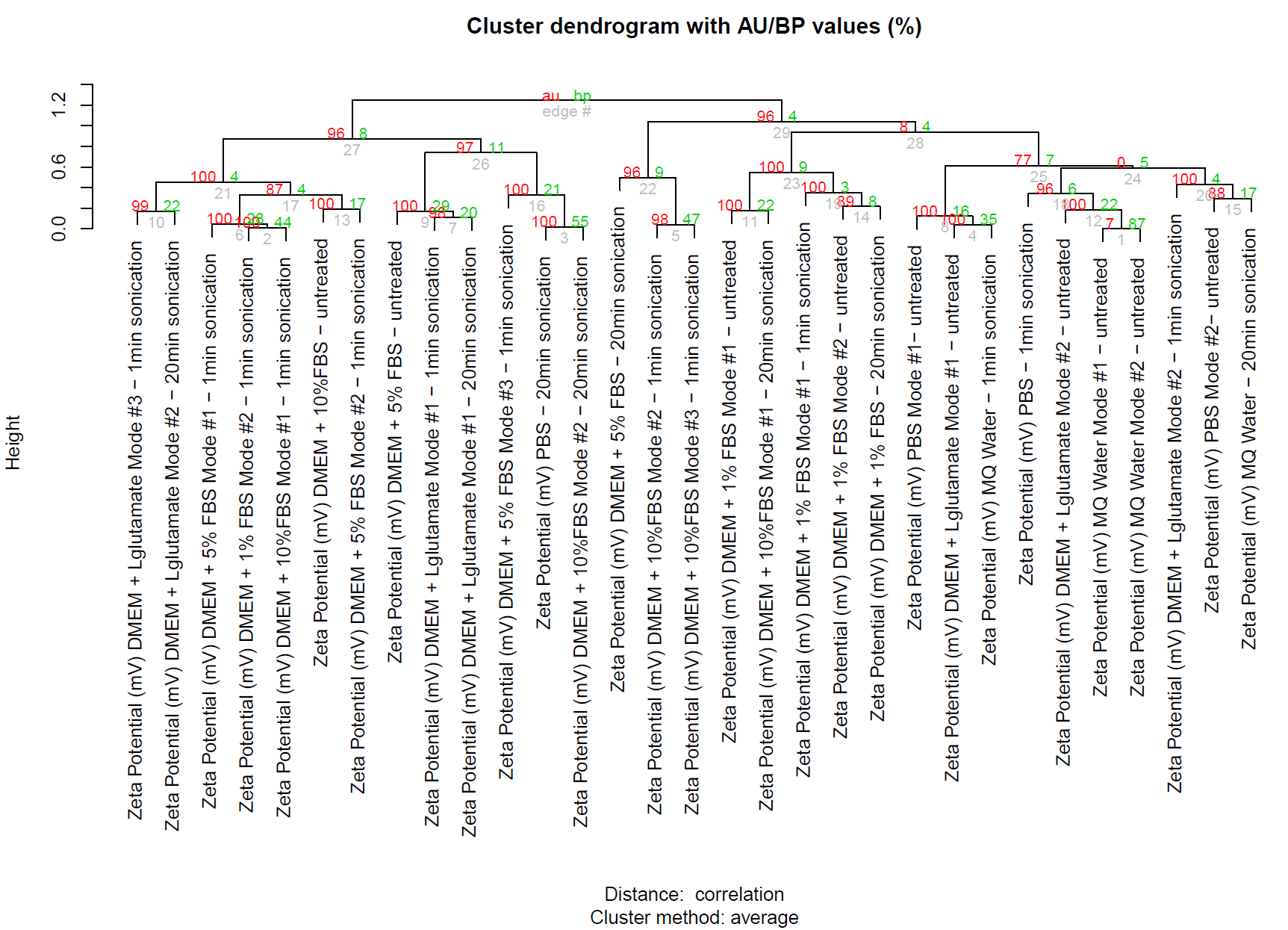


**Figure SM3.** Hierarchical clustering of the transposed matrix of Zeta Potential measures obtained for the nano-TiO_2_. Arrows indicate the medium-treatment values that were randomly selected for each cluster as representative.

### Polydispersibility Index (PdI)

Since the PdI data is obtained with the same instrument and test as the particle size distribution, the reader is referred to the particle size distribution section for further details. The hierarchical clustering of the transposed matrix of PdI with the randomly selected properties for each cluster is shown below.


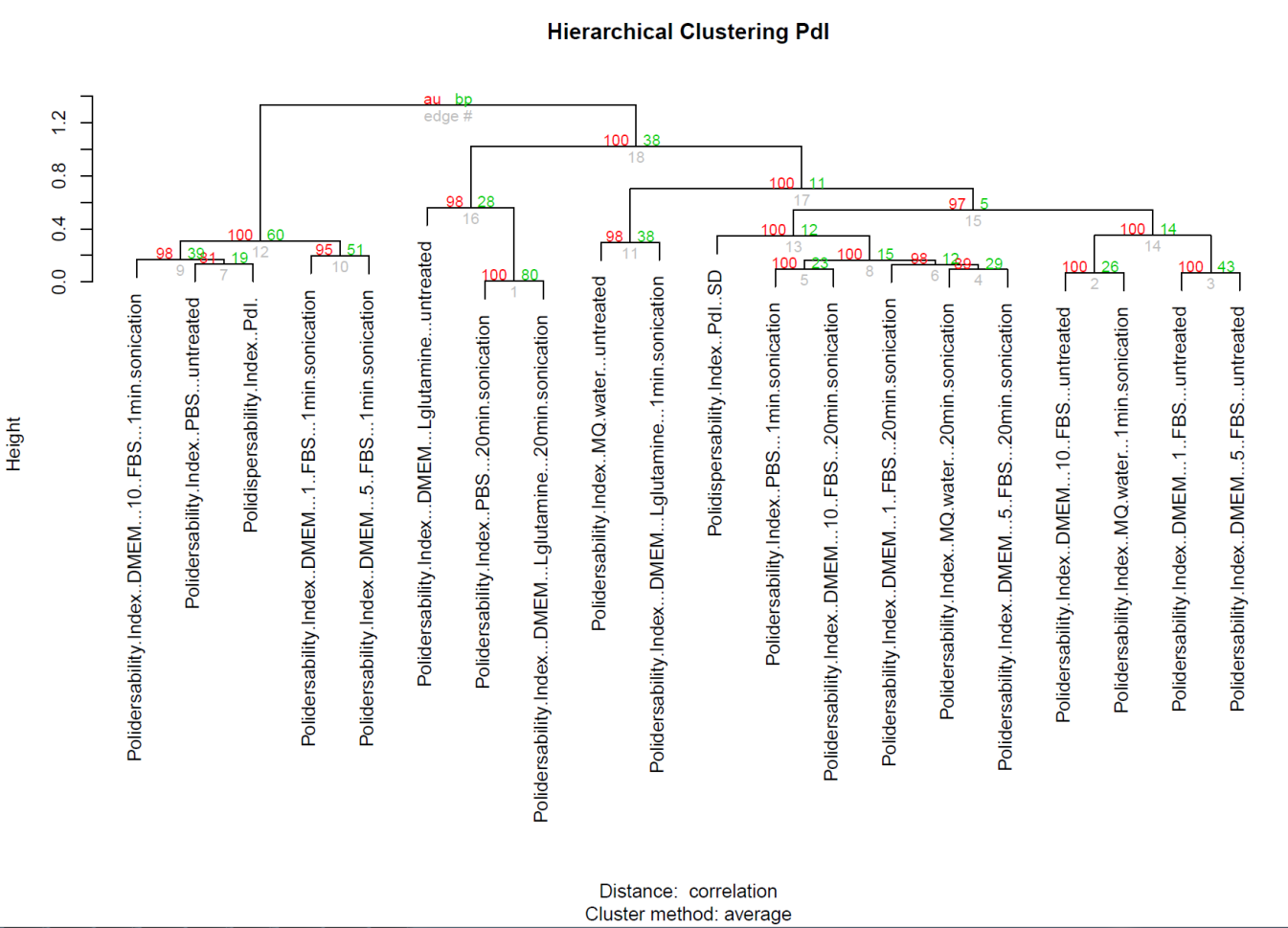


**Figure SM4.** Hierarchical clustering of the transposed matrix of polidispersibility index measures obtained for the nano-TiO_2_. Arrows indicate the medium-treatment values that were randomly selected for each cluster as representative.

### Isoelectric point

Isoelectric point corresponds to the pH-value at which the charge on the surface of the NM, i.e. Zeta Potential, is reversed. The data gathered was generated in different media (water and 0.1mM CaCl_2_), different conditions (acid-to-basic and basic-to-acid) and was found in three different sources (Nanogenotox, OECD dossiers, and in Cotogno et al. 2014). Some sources provided the exact values while in others the isoelectric point was given as an interval. In addition, no isoelectric point data could be found for NM-100. In order to take into account all these variations it was decided to consider the mean, minimum, and maximum isoelectric point data found for the NMs. This way, 3 rows with data relative to isoelectric point were added to the data matrix (see Table SM3).

**Table SM3.** Isoelectric point data included in the final dataset

| **Name** | **NM-100** | **NM-101** | **NM-102** | **NM-103** | **NM-104** | **NM-105** |
| --- | --- | --- | --- | --- | --- | --- |
| **Isoelectric Point (Mean) (pH)** | NA | 5.5 | 6.0 | 8.3 | 8.5 | 6.8 |
| **Isoelectric Point (Min) (pH)** | NA | 5.3 | 6.0 | 8.2 | 8.2 | 6.6 |
| **Isoelectric Point (Max) (pH)** | NA | 5.7 | 6 | 8.5 | 8.8 | 6.9 |

The fact that the nano-TiO_2_ dispersions appear to be unstable at pH 5-9 and that *in vitro* experiments are generally carried out at pH 7.4 indicate that the dispersions will tend to sediment, which might affect the results.

### Density

Density data was obtained from the OECD dossiers and the Nanogenotox project. Different methods were used to measure the density. Some of them declared the temperature at which the measure was carried out while others did not. Most of the methods rendered values of the order of 4.1 g/cm^3^ but two measures corresponded to 0.5-1.2 g/cm^3^ and 0.500-0.75 g/cm^3^ which are typical values of apparent density. The two latter values were discarded and the rest of the data was averaged.

### Porosity

Data on porosity was obtained solely from a single source and, therefore, there was no problem with the data treatment. Three different measures were found: total pore volume (ml/g), micro surface area (m^2^/g), and micropore volume (ml/g).

### Specific surface area

The data gathered for specific surface area corresponded to two different methods, SAXS and BET. The authors of Nanogenotox compared the two methods and observed a good agreement for values up to 130 m^2^/g. The values obtained for the same NM that were smaller than 130 m^2^/g were averaged. In the current dataset, only NM-101 was found to have values larger than 130 m^2^/g (316 and 169.5 m^2^/g for BET and SAXS, respectively). In such a situation, it is not recommended to average the values as they are not comparable. The fact that the BET values were closer to the values reported by the producers and that only BET values were available for NM-100 was the reason to select BET values over SAXS for NM-100 and NM-101..

### Dustiness (respirable)

Data on dustiness was obtained from the Nanogenotox project. Two methods, small rotating drum and the vortex shaker, were used and different parameters were measured, e.g. number of particles in 180s or 3600s, inhalable dustiness index, and respirable dustiness index. The authors compared the two methods and observed no significant correlation between them. In addition, they acknowledged that dustiness values depended on the characteristic properties of the powders and the activation energy in the simulated handling. Therefore different values may be obtained by the different test methods (test apparatus, operation procedure, sampling and measurement strategy, etc.). In the current case, only respirable dustiness index measured by small rotating drum was considered in the read-across because it was found to be the only method with data for all NMs.

### Biodurability

Data on biodurability corresponds to the amount of Ti, Al, and Si present in media after 24h of incubation of the NMs. Data on different media, i.e. water, bovine serum albumin 0.05%, Gambles solution, and Caco2 media was collected; but the data on water was discarded because all values were considered insoluble and data was not available for NM-100. The measures that were below the detection limit of the instrument were considered to be 0.

### Redox potential

The redox potential of NMs corresponds to the variation of O_2_ in the media during incubation. The data obtained from Nanogenotox was mainly qualitative and was transformed into 1 for oxidative behavior, -1 for reductive behavior, and 0 for neutral. Data in 3 different media (BSA, Gambles, and Caco2) was obtained.

## Full data matrix

**Table SM4.** Full dataset used for the nano-TiO_2_ read-across. Values highlighted in red correspond to monomodal distributions whose values have been used to populate the values of multimodal measures.

| ***Name*** | ***NM-100*** | ***NM-101*** | ***NM-102*** | ***NM-103*** | ***NM-104*** | ***NM-105*** |
| --- | --- | --- | --- | --- | --- | --- |
| ***In vitro* comet assay** | **1** | **0** | **1** | **0** | **0** | **1** |
| **Total non-TiO_2_ content including coating and impurities (% w/w)** | 1.5 | 9 | 5 | 11 | 11 | 0.11 |
| **Impurity (% w/w Fe)** | 0.49 | 0 | 0.07 | 0.06 | 0 | 0.06 |
| **Impurity (% w/w Si)** | 0.28 | 0.29 | 0.08 | 0.68 | 0.018 | 0.07 |
| **Impurity (% w/w K)** | 0.25 | 0 | 0.001 | 0.001 | 0.001 | 0 |
| **Impurity (% w/w P)** | 0.21 | 0.27 | 0.001 | 0 | 0 | 0 |
| **Impurity – coating (% w/w Al)** | 0.09 | 0.09 | 0.05 | 3.4 | 3.2 | 0.04 |
| **Impurity (% w/w Cr)** | 0.03 | 0 | 0 | 0 | 0 | 0 |
| **Impurity (% w/w Zr)** | 0.005 | 0.01 | 0.005 | 0.001 | 0.001 | 0 |
| **Impurity (% w/w Ca)** | 0.001 | 0 | 0.005 | 0.005 | 0.01 | 0 |
| **Impurity (% w/w Na)** | 0.001 | 0.1 | 0.001 | 0.01 | 0 | 0.001 |
| **Impurity (% w/w S)** | 0 | 0.22 | 0.001 | 0.01 | 0.01 | 0.26 |
| **Impurity (% w/w Mg)** | 0 | 0 | 0 | 0.001 | 0.001 | 0 |
| **Organic matter (% w/w)** | 0 | 8 | 0 | 2 | 2 | 0 |
| **Crystal type (Anatase)** | 1 | 1 | 1 | 0 | 0 | 0.84 |
| **Crystal type (Rutile)** | 0 | 0 | 0 | 1 | 1 | 0.16 |
| **Crystal type (Cubic)** | 0 | 0 | 0 | 0 | 0 | 0 |
| **Crystallite size (mean)** | 117.81 | 7.69 | 23.93 | 24.32 | 24.71 | 22.44 |
| **Surface coating (declared) (%)** | 0 | 0 | 0 | 8 | 8 | 0 |
| **Specific surface area (m^2^/g)** | 9.23 | 316.07 | 77.86 | 53.98 | 54.33 | 47.00 |
| **Shape (elongated=1, spherical=0)** | 0 | 0 | 0 | 1 | 0 | 1 |
| **Aspect ratio** | 1.53 | 1.53 | 1.53 | 1.70 | 1.53 | 1.36 |
| **Primary particle diameter (mean)** | 93.45 | 5.25 | 22.00 | 24.00 | 24.50 | 20.13 |
| **Particle size distribution (nm)** | 210 | 278 | 439.8 | 135.11 | 144.47 | 176.78 |
| **Particle size distribution-SD (nm)** | 10 | 0 | 36.66 | 25.27 | 35.21 | 38.99 |
| **Particle size distribution (Intensity distribution peak (nm))** | NA | NA | 685.55 | 146.62 | 193.94 | 180.75 |
| **Particle size distribution-SD (Intensity distribution peak (nm))** | NA | NA | 30.8 | 21.46 | 48.36 | 17.98 |
| **Particle size distribution (FWHM main peak)** | NA | NA | 444.5 | 82.36 | 100.52 | 75.47 |
| **Particle size distribution-SD (FWHM main peak)** | NA | NA | 94.9 | 12.74 | 31.37 | 10.20 |
| **Particle size distribution (after 24h) (nm)** | NA | NA | 969 | 198 | NA | 214 |
| **Particle size distribution-SD (after 24h) (nm)** | NA | NA | 7.65 | NA | NA | NA |
| **Particle size distribution in MQ Water, untreated, Mode #1 (nm)** | 391.2 | 1609 | 115 | 973.2 | 727.8 | 1102 |
| **Particle size distribution in MQ Water, untreated, Mode #2 (nm)** | 4862 | 1609 | 5170 | 973.2 | 727.8 | 204.7 |
| **Particle size distribution in PBS, untreated, Mode #1 (nm)** | 1440 | 1188 | 1528 | 1977 | 1817 | 4526 |
| **Particle size distribution in PBS, untreated, Mode #2 (nm)** | 5236 | 5148 | 5330 | 1977 | 5194 | 1150 |
| **Particle size distribution in DMEM + Lglutamate, untreated, Mode #1 (nm)** | 995.5 | 1438 | 2745 | 2255 | 3059 | 1881 |
| **Particle size distribution in DMEM + Lglutamate, untreated, Mode #2 (nm)** | 995.5 | 5560 | 2745 | 2255 | 3059 | 5372 |
| **Particle size distribution in DMEM + 1% FBS, untreated, Mode #1 (nm)** | 736 | 1201 | 1415 | 1040 | 1156 | 2454 |
| **Particle size distribution in DMEM + 1% FBS, untreated, Mode #2 (nm)** | 736 | 5232 | 1415 | 4593 | 5211 | 626.5 |
| **Particle size distribution in DMEM + 5% FBS, untreated, Mode #1 (nm)** | 845.4 | 1278 | 1414 | 991.1 | 719.3 | 1709 |
| **Particle size distribution in DMEM + 5% FBS, untreated, Mode #2 (nm)** | 845.4 | 1278 | 1414 | 991.1 | 5375 | 1709 |
| **Particle size distribution in DMEM + 10%FBS, untreated, Mode #1 (nm)** | 639.1 | 1406 | 1521 | 1156 | 711.2 | 1030 |
| **Particle size distribution in DMEM + 10%FBS, untreated, Mode #2 (nm)** | 4793 | 1406 | 1521 | 1156 | 711.2 | 4731 |
| **Particle size distribution in MQ water, untreated (nm)** | 343 | 1746 | 1062 | 671.6 | 367.8 | 720 |
| **Particle size distribution in PBS , untreated (nm)** | 2289 | 1229 | 1579 | 1397 | 1600 | 3342 |
| **Particle size distribution in DMEM + Lglutamine untreated (nm)** | 2129 | 1954 | 2427 | 1665 | 2869 | 2868 |
| **Particle size distribution in DMEM + 1% FBS, untreated (nm)** | 606.8 | 1166 | 1295 | 828.8 | 1111 | 1599 |
| **Particle size distribution in) DMEM + 5% FBS, untreated (nm)** | 621.3 | 1039 | 1234 | 653.2 | 657.5 | 1116 |
| **Particle size distribution in DMEM + 10% FBS, untreated (nm)** | 582.4 | 1127 | 1227 | 683.3 | 617.8 | 937.3 |
| **Particle size distribution in MQ Water, 1min sonication, Mode #1 (nm)** | 259.3 | 719.5 | 703 | 2649 | 207.7 | 352.6 |
| **Particle size distribution in MQ Water, 1min sonication, Mode #2 (nm)** | 259.3 | 719.5 | 703 | 2649 | 207.7 | 352.6 |
| **Particle size distribution in PBS, 1min sonication, Mode #1 (nm), Mode #1** | 2116 | 2254 | 2525 | 1629 | 4031 | 1682 |
| **Particle size distribution in PBS, 1min sonication, Mode #2 (nm)** | 2116 | 2254 | 2525 | 4619 | 465.2 | 5108 |
| **Particle size distribution in DMEM + Lglutamate, 1min sonication, Mode #1 (nm)** | 2973 | 2854 | 3488 | 4043 | 1701 | 4673 |
| **Particle size distribution in DMEM + Lglutamate, 1min sonication, Mode #2 (nm)** | 2973 | 2854 | 3488 | 4043 | 5560 | 1995 |
| **Particle size distribution in DMEM + 1% FBS , 1min sonication, Mode #1 (nm)** | 405.3 | 678.5 | 837.5 | 275.6 | 333.6 | 306.8 |
| **Particle size distribution in DMEM + 1% FBS, 1min sonication, Mode #2 (nm)** | 405.3 | 678.5 | 189.8 | 4344 | 4670 | 4755 |
| **Particle size distribution in DMEM + 5% FBS , 1min sonication, Mode #1 (nm)** | 408.8 | 755.5 | 901.8 | 432.4 | 278.2 | 336.9 |
| **Particle size distribution in DMEM + 5% , 1min sonication, Mode #2 (nm)** | 408.8 | 755.5 | 115.6 | 4881 | 278.2 | 4755 |
| **Particle size distribution in DMEM + 10%FBS, 1min sonication, Mode #1 (nm)** | 345.8 | 823.6 | 1077 | 370.9 | 334.4 | 349.8 |
| **Particle size distribution in DMEM + 10%FBS, 1min sonication, Mode #2 (nm)** | 345.8 | 823.6 | 1077 | 370.9 | 334.4 | 349.8 |
| **Particle size distribution in MQ water, 1min sonication (nm)** | 201.3 | 500.9 | 505.7 | 1977 | 194.3 | 227.5 |
| **Particle size distribution in PBS, 1min sonication (nm)** | 1624 | 1827 | 2079 | 2275 | 3197 | 3585 |
| **Particle size distribution in DMEM + , 1min sonication (nm)** | 2514 | 2350 | 2701 | 3551 | 3306 | 3507 |
| **Particle size distribution in DMEM + 1% FBS - 1min sonication) (nm)** | 310.4 | 521.2 | 590 | 263.5 | 278.5 | 265.3 |
| **Particle size distribution in DMEM + 5% FBS - 1min sonication) (nm)** | 315.2 | 569.2 | 617.3 | 345.8 | 225.8 | 286.3 |
| **Particle size distribution in DMEM + 10% FBS - 1min sonication) (nm)** | 283.9 | 623.4 | 732.2 | 286.9 | 267.8 | 281.2 |
| **Particle size distribution in MQ Water, 20min sonication, Mode #1 (nm)** | 378.8 | 1111 | 1103 | 765.3 | 344.5 | 902 |
| **Particle size distribution in MQ Water , 20min sonication, Mode #2 (nm)** | 378.8 | 4077 | 193.6 | 5041 | 4638 | 243.9 |
| **Particle size distribution in PBS , 20min sonication, Mode #1 (nm)** | 1042 | 1265 | 1789 | 1449 | 2779 | 4437 |
| **Particle size distribution in PBS Mode #2, 20min sonication, Mode #2 (nm)** | 5236 | 4976 | 4988 | 5037 | 2779 | 4437 |
| **Particle size distribution in DMEM + Lglutamate, 20min sonication, Mode #1 (nm)** | 1059 | 1974 | 2001 | 2916 | 3207 | 1956 |
| **Particle size distribution in DMEM + Lglutamate, 20min sonication, Mode #2 (nm)** | 1059 | 4881 | 5517 | 2916 | 3207 | 5290 |
| **Particle size distribution in DMEM + 1% FBS, 20min sonication, Mode #1 (nm)** | 631.9 | 1368 | 1063 | 684.1 | 975.4 | 969.6 |
| **Particle size distribution in DMEM + 1% FBS, 20min sonication, Mode #2 (nm)** | 5059 | 420.5 | 1063 | 4946 | 286.9 | 226.6 |
| **Particle size distribution in DMEM + 5% FBS, 20min sonication, Mode #1 (nm)** | 522.7 | 1073 | 1487 | 1079 | 925 | 848.3 |
| **Particle size distribution in DMEM + 5% FBS, 20min sonication, Mode #2 (nm)** | 5017 | 5046 | 1487 | 1079 | 925 | 4864 |
| **Particle size distribution in DMEM + 10%FBS, 20min sonication, Mode #1 (nm)** | 565.7 | 1255 | 1228 | 1155 | 605.2 | 1110 |
| **Particle size distribution in DMEM + 10%FBS, 20min sonication, Mode #2 (nm)** | 565.7 | 5222 | 1228 | 262.3 | 4991 | 1110 |
| **Particle size distribution in MQ water - 20min sonication)** | 307.6 | 1130 | 794.1 | 596.9 | 290.8 | 474.4 |
| **Particle size distribution in PBS,- 20min sonication (nm)** | 1217 | 1276 | 1809 | 1350 | 2284 | 4514 |
| **Particle size distribution in DMEM + Lglutamine, 20min sonication (nm)** | 1754 | 1992 | 1997 | 2268 | 2636 | 2938 |
| **Particle size distribution in DMEM + 1% FBS, 20min sonication (nm)** | 540.2 | 668.7 | 975.4 | 526.8 | 520.4 | 743.9 |
| **Particle size distribution in DMEM + 5% FBS, 20min sonication (nm)** | 450.4 | 1065 | 1197 | 656.9 | 696.2 | 921.8 |
| **Particle size distribution in DMEM + 10% FBS, 20min sonication (nm)** | 473.1 | 957.9 | 874.8 | 570.3 | 480.8 | 619.8 |
| **Zeta Potential in MQ Water, 1min sonication (mV)** | -24.5 | -27.2 | -27.1 | 39.1 | -23.4 | -23.8 |
| **Zeta Potential in PBS, 1min sonication (mV)** | -26.7 | -19.7 | -25.1 | -20.8 | -16.9 | -20.5 |
| **Zeta Potential in DMEM + Lglutamate, 1min sonication, Mode #1 (mV)** | 20.5 | 22.3 | -3.14 | -8.44 | -7.29 | -2.55 |
| **Zeta Potential in DMEM + Lglutamate, 1min sonication, Mode #2 (mV)** | -26 | -34.3 | -3.14 | -8.44 | -7.29 | -2.55 |
| **Zeta Potential in DMEM + Lglutamate, 1min sonication, Mode #3 (mV)** | 95.4 | -92 | -3.14 | -8.44 | -7.29 | -2.55 |
| **Zeta Potential in DMEM + 1% FBS, 1min sonication, Mode #1 (mV)** | -9.14 | -11.8 | -13.6 | -9.98 | -8.88 | -9.37 |
| **Zeta Potential in DMEM + 1% FBS, 1min sonication, Mode #2 (mV)** | 140 | -11.8 | -13.6 | -9.98 | -8.88 | -9.37 |
| **Zeta Potential in DMEM + 5% FBS, 1min sonication, Mode #1 (mV)** | 107 | -15 | -13.4 | -12 | 15.1 | 9.43 |
| **Zeta Potential in DMEM + 5% FBS, 1min sonication, Mode #2 (mV)** | 35.4 | -15 | -13.4 | -12 | -43.7 | -47.9 |
| **Zeta Potential in DMEM + 5% FBS, 1min sonication, Mode #3 (mV)** | -21.2 | -15 | -13.4 | -12 | -43.7 | -47.9 |
| **Zeta Potential in DMEM + 10%FBS, 1min sonication, Mode #1 (mV)** | 78.4 | 0.13 | -10.5 | -12.4 | -9.38 | -9.92 |
| **Zeta Potential in DMEM + 10%FBS, 1min sonication, Mode #2 (mV)** | 12.2 | 0.13 | -10.5 | -12.4 | 129 | -9.92 |
| **Zeta Potential in DMEM + 10%FBS, 1min sonication, Mode #3 (mV)** | -27.1 | 0.13 | -10.5 | -12.4 | 129 | -9.92 |
| **Zeta Potential in MQ Water - 20min sonication (mV)** | -40.6 | -27.5 | 30.3 | 39.1 | 24.6 | -32.6 |
| **Zeta Potential in PBS - 20min sonication (mV)** | -20.2 | -21.7 | -18.5 | -20.9 | -20.3 | -33.2 |
| **Zeta Potential in DMEM + Lglutamate, 20min sonication, Mode #1 (mV)** | -1.55 | 3.6 | -3.46 | -8.76 | -9.98 | -8.55 |
| **Zeta Potential in DMEM + Lglutamate, 20min sonication, Mode #2 (mV)** | -1.55 | -42.5 | -3.46 | -8.76 | -9.98 | -8.55 |
| **Zeta Potential in DMEM + 1% FBS, 20min sonication (mV)** | -11.4 | -12 | -12.4 | -10 | -10.2 | -7.76 |
| **Zeta Potential in DMEM + 5% FBS, 20min sonication (mV)** | -10.4 | -11.3 | -9.47 | -13.7 | -9.38 | -11.9 |
| **Zeta Potential in DMEM + 10%FBS, 20min sonication, Mode #1 (mV)** | -11.3 | -11.5 | -10.4 | -11.8 | -10.5 | -5.43 |
| **Zeta Potential in DMEM + 10%FBS, 20min sonication, Mode #2 (mV)** | -11.3 | -11.5 | -10.4 | -11.8 | -10.5 | -74 |
| **Polydispersity Index in MQ water - untreated** | 0.205 | 0.264 | 0.187 | 0.287 | 0.376 | 0.376 |
| **Polydispersity Index in PBS - untreated** | 0.219 | 0.239 | 0.769 | 0.255 | 0.232 | 0.232 |
| **Polydispersity Index in DMEM + Lglutamine – untreated** | 0.332 | 0.359 | 0.181 | 0.256 | 0.247 | 0.247 |
| **Polydispersity Index in DMEM + 1% FBS - untreated** | 0.207 | 0.201 | 0.081 | 0.269 | 0.208 | 0.208 |
| **Polydispersity Index in DMEM + 5% FBS - untreated** | 0.194 | 0.232 | 0.139 | 0.293 | 0.22 | 0.22 |
| **Polydispersity Index in DMEM + 10% FBS - untreated** | 0.176 | 0.194 | 0.182 | 0.369 | 0.201 | 0.201 |
| **Polydispersity Index in MQ water - 1min sonication** | 0.205 | 0.274 | 0.248 | 0.393 | 0.236 | 0.211 |
| **Polydispersity Index in PBS - 1min sonication** | 0.219 | 0.283 | 0.188 | 0.442 | 0.334 | 0.443 |
| **Polydispersity Index in DMEM + Lglutamine - 1min sonication** | 0.332 | 0.217 | 0.268 | 0.279 | 0.434 | 0.395 |
| **Polydispersity Index in DMEM + 1% FBS - 1min sonication** | 0.207 | 0.232 | 0.243 | 0.243 | 0.194 | 0.177 |
| **Polydispersity Index in DMEM + 5% FBS - 1min sonication** | 0.194 | 0.232 | 0.27 | 0.25 | 0.161 | 0.207 |
| **Polydispersity Index in DMEM + 10% FBS - 1min sonication** | 0.176 | 0.24 | 0.27 | 0.196 | 0.178 | 0.196 |
| **Polydispersity Index in MQ water - 20min sonication** | 0.199 | 0.351 | 0.254 | 0.393 | 0.306 | 0.443 |
| **Polydispersity Index in PBS - 20min sonication** | 0.317 | 0.238 | 0.231 | 0.25 | 0.227 | 0.274 |
| **Polydispersity Index in DMEM + Lglutamine - 20min sonication** | 0.515 | 0.247 | 0.227 | 0.264 | 0.209 | 0.341 |
| **Polydispersibility Index in DMEM + 1% FBS - 20min sonication** | 0.195 | 0.282 | 0.054 | 0.317 | 0.282 | 0.48 |
| **Polydispersibility Index in DMEM + 5% FBS - 20min sonication** | 0.223 | 0.302 | 0.179 | 0.367 | 0.221 | 0.456 |
| **Polydispersibility Index in DMEM + 10% FBS - 20min sonication** | 0.204 | 0.234 | 0.235 | 0.417 | 0.239 | 0.391 |
| **Polydispersity Index (PdI)** | NA | 0.323 | 0.427 | 0.292 | 0.227 | 0.245 |
| **IsoelectricPoint (Mean)** | NA | 5.5 | 6 | 8.3 | 8.5 | 6.8 |
| **IsoelectricPoint (Min)** | NA | 5.3 | 6 | 8.2 | 8.2 | 6.6 |
| **IsoelectricPoint (Max)** | NA | 5.7 | 6 | 8.5 | 8.8 | 6.9 |
| **Density (g/mL)** | 3.84 | 3.99 | 3.84 | 4.015 | 4.09 | 4.052 |
| **Mean of total pore volume (mL/g)** | 0.0324 | 0.319 | 0.2996 | 0.2616 | 0.1935 | 0.1937 |
| **Micro surface area (m^2^/g)** | 0 | 13.625 | 1.108 | 0 | 0 | 0 |
| **Micropore volume (mL/g)** | 0 | 0.00179 | 0.00034 | 0 | 0 | 0 |
| **Specific surface area (mean)** | 9.23 | 242.785 | 77.864 | 53.983 | 54.331 | 47 |
| **Dustiness-Respirable(mg/kg)** | 1500 | 5600 | 9200 | 19000 | 6400 | 11000 |
| **Biodurability 24h 0.05% BSA (Ti content) (µg/l)** | 5.2 | 0 | 0 | 0 | 0 | 0 |
| **Biodurability 24h Gambles solution (Ti content) (µg/l)** | 0 | 0 | 3388 | 0 | 0 | 0 |
| **Biodurability 24h Caco2 (Ti content) (µg/l)** | 796 | 3414 | 1741 | 222 | 3386 | 2724 |
| **Biodurability 24h 0.05% BSA (Al content) (µg/l)** | 0 | 175 | 0 | 198 | 137 | 0 |
| **Biodurability 24h Gambles solution (Al content) (µg/l)** | 0 | 177 | 0 | 868 | 413 | 0 |
| **Biodurability 24h Caco2 (Al content) (µg/l)** | 24 | 252 | 0 | 182 | 413 | 0 |
| **Biodurability 24h 0.05% BSA (Si content) (mg/l)** | 0 | 0 | 0 | 0.9 | 0 | 0 |
| **Biodurability 24h Gambles solution (Si content) (µg/l)** | 0 | 0 | 0 | 2.0 | 0 | 0 |
| **Biodurability 24h Caco2 (Si content) (µg/l)** | 0 | 0 | 0 | 1.7 | 0 | 0 |
| **Redox Caco2 medium** ^Ω^ | 1 | -1 | -1 | 1 | -1 | -1 |
| **Redox Gamble's solution** ^Ω^ | 1 | 0 | -1 | 1 | -1 | -1 |
| **Redox BSA** ^Ω^ | 0 | 0 | 0 | 0 | 0 | 0 |

^Ω^ values obtained from Nanogenotox 4.7 determined by measuring the content of O_2_. Oxidising properties (1), neutral (0), reducing (-1)

## Literature search on genotoxicity studies

Our initial dataset on toxicological endpoints was collected from the OECD dossier on TiO_2_ (OECD 2015) that, although not aimed specifically at hazard assessment, is considered an updated NMs data repository. This toxicological dataset was expanded for the selected endpoint to be read across (genotoxicity) by searching available studies in the literature.

A bibliographic search was done in August 2016 in Scopus using the keywords genotox*, nano*, TiO_2_. It resulted in 152 review and research papers. A first selection was done depending on title and abstract contents: if it was not about genotoxicity testing, the papers were not included. Also, only toxicity studies relevant for human health hazard assessment were considered (studies on bacteria, plants, mussels, fish were excluded).

A reliability assessment of the collected studies was performed according to the criteria defined by (ANSES, 2016), which states that reliable studies must contain:

1. NM's characterisation (at least size, crystallinity and coating) and a description of the dispersed materials (particle size distribution, zeta potential, polydispersity index)
2. Observed NM uptake and/or non-cytotoxicity
3. Positive and negative controls as well as replicates

Micronucleus, comet and chromosomal aberration tests were collected because the current OECD test guidelines for these tests are considered applicable to NMs. The *in vitro* comet assay was selected for the read-across case study because it was the only one with data for the 6 source analogues.

Table SM5 reports the list of papers containing genotoxicity studies that were taken into consideration for the selection of the genotoxicity tests to read across. According to the first criteria, related to NMs characterisation, the information necessary for identification was available in the reliable studies, hence the studied NM could be assigned to the corresponding analogue in our case study. The results coming from the reliable studies reported in the table are accounted for in Table 2 in the main text.

**Table SM5.** List of genotoxicity studies on TiO_2_ found in the literature with the corresponding reliability call assigned according to (ANSES 2016) criteria

|  | **Assay type** | **Reference** | **Reliability *** |
| --- | --- | --- | --- |
| *In vivo* | Comet | (Dobrzyńska et al. 2014) | Unreliable |
|  |  | (Trouiller et al. 2009) | Unreliable |
|  |  | (Suzuki et al. 2016) | Unreliable |
|  |  | (Louro et al. 2014) | Reliable |
|  | Micronucleus | (Dobrzyńska et al. 2014) | Unreliable |
|  |  | (Trouiller et al. 2009) | Unreliable |
|  |  | (Suzuki et al. 2016) | Unreliable |
|  |  | (Louro et al. 2014) | Reliable |
|  | Chromosome aberration | (Louro et al. 2014) | Reliable |
| *In vitro* | Comet | (Prasad et al. 2013) | Reliable |
|  |  | (Vales et al. 2014) | Reliable |
|  |  | (Kansara et al. 2015) | Reliable |
|  |  | (Armand et al. 2016) | Reliable |
|  |  | (Jugan et al. 2012) | Reliable |
|  |  | (Gerloff et al. 2012) | Reliable |
|  |  | (Kermanizadeh et al. 2012) | Reliable |
|  |  | (Kermanizadeh et al. 2013) | Reliable |
|  |  | (Stoccoro et al. 2016) | Unreliable |
|  |  | (Guichard et al. 2012) | Reliable |
|  |  | (Barillet et al. 2010) | Reliable |
|  |  | (Guichard et al. 2012) | Reliable |
|  | Micronucleus | (Prasad et al. 2013) | Reliable |
|  |  | (Prasad et al. 2014) | Reliable |
|  |  | (Vales et al. 2014) | Reliable |
|  |  | (Kansara et al. 2015) | Reliable |
|  |  | (Jugan et al. 2012) | Reliable |
|  |  | (Armand et al. 2016) | Reliable |
|  |  | (Tavares et al. 2014) | Reliable |
|  |  | (Stoccoro et al. 2016) | Unreliable |
|  |  | (Guichard et al. 2012) | Reliable |
|  | Chromosome aberration | (L Browning et al. 2014) | Reliable |

*Reliability of the studies was assessed following the criteria reported in the main text.

## List of the genotoxicity studies taken into consideration

Armand, L., A. Tarantini, D. Beal, M. Biola-Clier, L. Bobyk, S. Sorieul, K. Pernet-Gallay, C. Marie-Desvergne, I. Lynch, N. Herlin-Boime, and M. Carriere. (2016). Long-term exposure of A549 cells to titanium dioxide nanoparticles induces DNA damage and sensitizes cells towards genotoxic agents. Nanotoxicology 10:913–923.

Barillet, S., A. Simon-Deckers, N. Herlin-Boime, M. Mayne-L’Hermite, C. Reynaud, D. Cassio, B. Gouget, and M. Carrière. (2010). Toxicological consequences of TiO2, SiC nanoparticles and multi-walled carbon nanotubes exposure in several mammalian cell types: an in vitro study. Journal of Nanoparticle Research 12:61–73.

Dobrzyńska, M. M., A. Gajowik, J. Radzikowska, A. Lankoff, M. Dušinská, and M. Kruszewski. (2014). Genotoxicity of silver and titanium dioxide nanoparticles in bone marrow cells of rats in vivo. Toxicology 315:86–91.

Gerloff, K., I. Fenoglio, E. Carella, J. Kolling, C. Albrecht, A. W. Boots, I. Förster, and R. P. F. Schins. (2012). Distinctive Toxicity of TiO2 Rutile/Anatase Mixed Phase Nanoparticles on Caco-2 Cells.

Guichard, Y., J. Schmit, C. Darne, L. Gaté, M. Goutet, D. Rousset, O. Rastoix, R. Wrobel, O. Witschger, A. Martin, V. Fierro, and S. Binet. (2012). Cytotoxicity and genotoxicity of nanosized and microsized titanium dioxide and iron oxide particles in Syrian hamster embryo cells. The Annals of occupational hygiene 56:631–44.

Jugan, M.-L., S. Barillet, A. Simon-Deckers, N. Herlin-Boime, S. Sauvaigo, T. Douki, and M. Carriere. (2012). Titanium dioxide nanoparticles exhibit genotoxicity and impair DNA repair activity in A549 cells. Nanotoxicology 6:501–513.

Kansara, K., P. Patel, D. Shah, R. K. Shukla, S. Singh, A. Kumar, and A. Dhawan. (2015). TiO 2 nanoparticles induce DNA double strand breaks and cell cycle arrest in human alveolar cells. Environmental and Molecular Mutagenesis 56:204–217.

Kermanizadeh, A., G. Pojana, B. K. Gaiser, R. Birkedal, D. Bilanicova, H. Wallin, K. A. Jensen, B. Sellergren, G. R. Hutchison, A. Marcomini, and V. Stone. (2012). In vitro assessment of engineered nanomaterials using C3A cells: Cytotoxicity, pro-inflammatory cytokines and function markers. Nanotoxicology 5390.

Kermanizadeh, A., S. Vranic, S. Boland, K. Moreau, A. Baeza-Squiban, B. K. Gaiser, L. A. Andrzejczuk, and V. Stone. (2013). An in vitro assessment of panel of engineered nanomaterials using a human renal cell line: cytotoxicity, pro-inflammatory response, oxidative stress and genotoxicity. BMC Nephrology 14:96.

L Browning, C., T. The, and M. D. M. and J. P. W. Sr. (2014). Titanium Dioxide Nanoparticles are not Cytotoxic or Clastogenic in Human Skin Cells. Journal of Environmental & Analytical Toxicology 4.

Louro, H., A. Tavares, N. Vital, P. M. Costa, E. Alverca, E. Zwart, W. H. de Jong, V. Fessard, J. Lavinha, and M. J. Silva. (2014). Integrated approach to the in vivo genotoxic effects of a titanium dioxide nanomaterial using LacZ plasmid-based transgenic mice. Environmental and Molecular Mutagenesis 55:500–509.

OECD. (2015). Titanium dioxide dossier (NM100-NM105). ENV/CHEM/NANO(2015)21/Part 1 to 5.

Prasad, R. Y., S. O. Simmons, M. G. Killius, R. M. Zucker, A. D. Kligerman, C. F. Blackman, R. C. Fry, and D. M. DeMarini. (2014). Cellular interactions and biological responses to titanium dioxide nanoparticles in HepG2 and BEAS-2B cells: Role of cell culture media. Environmental and Molecular Mutagenesis 55:336–342.

Prasad, R. Y., K. Wallace, K. M. Daniel, A. H. Tennant, R. M. Zucker, J. Strickland, K. Dreher, A. D. Kligerman, C. F. Blackman, and D. M. Demarini. (2013). Effect of treatment media of titanium dioxide nanoparticles: impact on genotoxicity, cellular interaction, and cell cycle. ACS Nano 7:1929–1942.

Stoccoro, A., S. Di Bucchianico, C. Uboldi, F. Coppedè, J. Ponti, C. Placidi, M. Blosi, S. Ortelli, A. L. Costa, and L. Migliore. (2016). A panel of in vitro tests to evaluate genotoxic and morphological neoplastic transformation potential on Balb/3T3 cells by pristine and remediated titania and zirconia nanoparticles. Mutagenesis.

Suzuki, T., N. Miura, R. Hojo, Y. Yanagiba, M. Suda, T. Hasegawa, M. Miyagawa, and R.-S. Wang. (2016). Genotoxicity assessment of intravenously injected titanium dioxide nanoparticles in gpt delta transgenic mice. Mutation Research/Genetic Toxicology and Environmental Mutagenesis 802:30–37.

Tavares, A. M., H. Louro, S. Antunes, S. Quarré, S. Simar, P.-J. De Temmerman, E. Verleysen, J. Mast, K. A. Jensen, H. Norppa, F. Nesslany, and M. J. Silva. (2014). Genotoxicity evaluation of nanosized titanium dioxide, synthetic amorphous silica and multi-walled carbon nanotubes in human lymphocytes. Toxicology in Vitro 28:60–69.

Trouiller, B., R. Reliene, A. Westbrook, P. Solaimani, and R. H. Schiestl. (2009). Titanium dioxide nanoparticles induce DNA damage and genetic instability in vivo in mice. Cancer research 69:8784–9.

Vales, G., L. Rubio, and R. Marcos. (2014). Long-term exposures to low doses of titanium dioxide nanoparticles induce cell transformation, but not genotoxic damage in BEAS-2B cells. Nanotoxicology 9:568–578.
